# Silver Coordination Polymers Driven by Adamantoid Blocks for Advanced Antiviral and Antibacterial Biomaterials

**DOI:** 10.1021/acsami.3c15606

**Published:** 2024-03-08

**Authors:** Sabina W. Jaros, Magdalena Florek, Barbara Bażanów, Jarosław Panek, Agnieszka Krogul-Sobczak, M. Conceição Oliveira, Jarosław Król, Urszula Śliwińska-Hill, Dmytro S. Nesterov, Alexander M. Kirillov, Piotr Smoleński

**Affiliations:** † Faculty of Chemistry, University of Wrocław, F. Joliot-Curie 14, 50-383 Wrocław, Poland; ‡ Department of Veterinary Microbiology, Wrocław University of Environmental and Life Sciences, Norwida 31, 50-375 Wrocław, Poland; § Faculty of Chemistry, University of Warsaw, Pasteura 1, 02-093 Warsaw, Poland; ∥ Centro de Química Estrutural, Institute of Molecular Sciences, Departamento de Engenharia Química, Instituto Superior Técnico, Universidade de Lisboa, Av. Rovisco Pais, 1049-001 Lisbon, Portugal; ⊥ Faculty of Pharmacy, Department of Basic Chemical Sciences, Wrocław Medical University, Borowska 211, 50-566 Wrocław, Poland

**Keywords:** antiviral materials, antibacterial biopolymers, silver compounds, coordination polymers, hybrid starch-based materials, personal health-protection materials

## Abstract

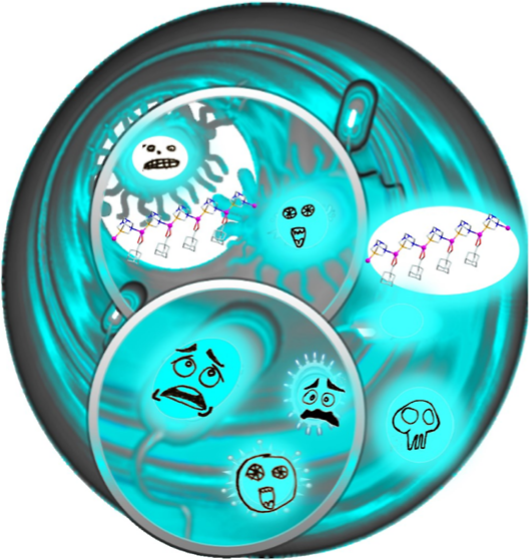

The development of sustainable biomaterials and surfaces to prevent the accumulation and proliferation of viruses and bacteria is highly demanded in healthcare areas. This study describes the assembly and full characterization of two new bioactive silver(I) coordination polymers (CPs) formulated as [Ag(aca)(μ-PTA)]_*n*_·5*n*H_2_O (**1**) and [Ag_2_(μ-ada)(μ_3_-PTA)_2_]_*n*_·4*n*H_2_O (**2**). These products were generated by exploiting a heteroleptic approach based on the use of two different adamantoid building blocks, namely 1,3,5-triaza-7-phosphaadamantane (PTA) and 1-adamantanecarboxylic (Haca) or 1,3-adamantanedicarboxylic (H_2_ada) acids, resulting in the assembly of 1D (**1**) and 3D (**2**). Antiviral, antibacterial, and antifungal properties of the obtained compounds were investigated in detail, followed by their incorporation as bioactive dopants (1 wt %) into hybrid biopolymers based on acid-hydrolyzed starch polymer (AHSP). The resulting materials, formulated as **1**@AHSP and **2**@AHSP, also featured (i) an exceptional antiviral activity against herpes simplex virus type 1 and human adenovirus (HAd-5) and (ii) a remarkable antibacterial activity against Gram-negative bacteria. Docking experiments, interaction with human serum albumin, mass spectrometry, and antioxidation studies provided insights into the mechanism of antimicrobial action. By reporting these new silver CPs driven by adamantoid building blocks and the derived starch-based materials, this study endows a facile approach to access biopolymers and interfaces capable of preventing and reducing the proliferation of a broad spectrum of different microorganisms, including bacteria, fungi, and viruses.

## Introduction

Microbial contamination, especially of bacterial and viral origin, tremendously impacts our everyday life.^1−3^ Resistance of microorganisms to conventional disinfecting and therapeutic agents and their ability to produce biofilms represent severe global issues.^1−3^ In particular, there is an alarming problem of the reduced susceptibility of many clinically important pathogens, including members of *Enterobacterales*, *Pseudomonas aeruginosa* (*P. aeruginosa*), and *Staphylococcus aureus* (*S. aureus*), which explains the urgent need for new antibacterial agents and disinfectants.^1−3^ Viral infections carry a risk of severe illness and even death, of which the recent COVID-19 pandemic is a stark example.^4^ Moreover, viruses such as human adenoviruses 36 (HAdV-36) and 5 (HAdV-5) can cause infectious obesity.^5^ Therefore, new advanced materials capable of combating viruses and resistant bacteria, and averting the growth of surface-attached microorganisms are currently in high demand.^6−9^ Application of surface-active antimicrobials to prevent the proliferation of microorganisms has received increasing research attention.^7,8,10^ As simple and sustainable materials, starch biopolymers doped with metal-based biocides are particularly promising. Such materials are ideal for creating antimicrobial packaging/surfaces, microbe-resistant materials, and personal healthcare products, which can also fulfill sustainability challenges.^7−12^ Starch is an inexpensive and tunable natural product that finds wide applications in the food and biotechnology industries.^8^ Depending on the plant from which starch is extracted, the polysaccharide ratio (amylose/amylopectin) may significantly vary and provide unique chemical behavior. Although starch alone does not exhibit antimicrobial activity, there are many research studies on the development of efficient starch-based materials with desired targeted antibacterial action, biodegradability, and physical and release properties.^7−13^ For example, Abreu et al. reported a series of antibacterial starch-based materials with/or without silver additives,^10^ which were active against *S. aureus* and *Escherichia coli* (*E. coli*) strains. However, these materials were not active against yeast such as *Candida albicans* (*C. albicans*), which limits their application in food packaging.^10^ Ortega et al. demonstrated that antibacterial starch-based films containing silver nanoparticles can increase the freshness of different products.^12^ A significant advantage of starch-based materials is their biodegradability and tailorable delivery of bioactive components. This is related to the capability of chemical derivatization, including the doping with bioactive coordination polymers (CPs).^7−13^ Hydrolysis of starch-based films is a way of aging and raising their solubility (biodegradability) level as a postsynthesis treatment with an acid or enzymes.^13^ However, such postsynthetic treatment can lead to the decomposition of biologically active CP-based additives, including those containing silver ions. Moreover, the antibacterial action and rate of silver ion release are closely related to the polymeric matrix in which they are located. Hybrid Ag-doped films based on epoxidized soybean oil acrylate revealed lower antibacterial activity than the potato starch-based films loaded with the same silver compounds.^6^ This is related to the type of matrix and its biodegradability/solubility, thus directly affecting the silver ion release profile.

We observed that an effective approach to minimize the possibility of silver reduction and to accelerate silver ion release consists of a directed in situ hydrolysis of starch and a controlled aging acceleration just before merging with Ag-CPs. This approach leads to the generation of novel and readily biodegradable acid-hydrolyzed starch polymer (AHSP), which can be used as a matrix for antibacterial and antiviral dopants based on silver(I) CPs. Such AHSP-based composites can find potential applications in antimicrobial pads for oral hygiene devices, such as irrigators or electric toothbrushes. Due to the typical location of these devices in bathrooms or toilets, they are exposed to contamination by fecal bacteria (e.g., *E. coli*). On the other hand, these devices may transmit to surfaces some pathogens, such as herpes simplex virus type 1 (HSV-1) and human adenovirus (HAd-5). Therefore, such starch-based antimicrobial pads should contain dopants with prominent antiviral, antifungal, and antibacterial efficiency. In turn, the antimicrobial efficiency can be enhanced by exploring a heteroleptic approach through the synergistic effect of different bioactive components.

Among the parameters that affect the biological activity of silver-based CPs, those that matter the most are the metal coordination environment, lipo- and hydrophilicity, and the number and type of bioactive organic building blocks.^6,14,15^ Ligands originating from adamantane derivatives represent a group of attractive building blocks that exhibit various therapeutic properties (e.g., antiviral, antibacterial, antiparasitic, anticancer, and anti-inflammatory).^16^ Such compounds can also act as angiogenesis inhibitors^16^ and hypoglycemic agents for treating type 2 diabetes,^17,18^ insulin-dependent diabetes, and obesity.^19−21^ Nonetheless, the ligands with adamantane-like cores are still little explored as building blocks for generating CPs, especially those incorporating silver(I) ions.

Following our interest in assembling novel types of bioactive metal–organic architectures based on aminophosphine and carboxylate ligands,^22−24^ this work describes the synthesis and characterization of two new examples of silver(I) CPs, namely [Ag(aca)(μ-PTA)]_*n*_·5*n*H_2_O (**1**) and [Ag_2_(μ-ada)(μ_3_-PTA)_2_]_*n*_·4*n*H_2_O (**2**). These compounds were assembled via a heteroleptic approach based on the use of two adamantane-like building blocks, namely 1,3,5-triaza-7-phosphaadamantane (PTA) and 1-adamantanecarboxylic (Haca) or 1,3-adamantanedicarboxylic (H_2_ada) acids. Because of prominent broad-spectrum antimicrobial effectiveness related to the synergistic action of all used components, the obtained compounds **1** and **2** were used as bioactive dopants (1 wt %) for the generation of antiviral and antibacterial surfaces, **1**@AHSP and **2**@AHSP, derived from AHSP. This study thus also describes the preparation, characterization, and antibacterial and antiviral properties of hybrid biopolymer films, which endow a new approach for the design of multifunctional materials and surfaces capable of preventing the proliferation of different types of microorganisms.

## Results and Discussion

### Synthesis of CPs

Two new silver(I) CPs were assembled by combining two types of adamantane-like derivatives in each structure. The synthetic approach is based on a mixed-ligand synthesis undergoing in a water/methanol medium under ambient conditions.^22−24^ The microcrystalline solids of isolated compounds [Ag(aca)(μ-PTA)]_*n*_·5*n*H_2_O (**1**) and [Ag_2_(μ-ada)(μ_3_-PTA)_2_]_*n*_·4*n*H_2_O (**2**) were characterized by elemental analysis, powder X-ray diffraction (PXRD) (Figures S1 and S2), Fourier transform infrared (FTIR) (Figures S3 and S4), and nuclear magnetic resonance (NMR) (Figures S5 and S6) spectroscopy. The crystal structures were established by single-crystal X-ray diffraction that revealed 1D (**1**) and 3D (**2**) CP networks. The purity of the bulk samples of **1** and **2** was attested by PXRD that revealed a good match between the experimental and calculated patterns.

In the carboxylate region of the FTIR spectra of **1** and **2**, a set of characteristic symmetric and asymmetric vibrations of deprotonated adamantane-based carboxylate ligands was identified. The calculated frequency difference indicates the chelating coordination mode of COO^–^ groups (Δν = 143 cm^–1^ for **1** and 152 cm^–1^ for **2**), which is further confirmed by crystallographic data. The NMR spectra of **1** and **2** in D_2_O also show characteristic signals ensuring the coordination of both types of adamantane-based ligands. It should be noted that both compounds exhibit some solubility in water with the *S*_25 °C_ values of 4.5 (**1**) and 2.0 (**2**) mg·mL^–1^.

### Crystal Structures of CPs **1** and **2**

**Figure 1:**
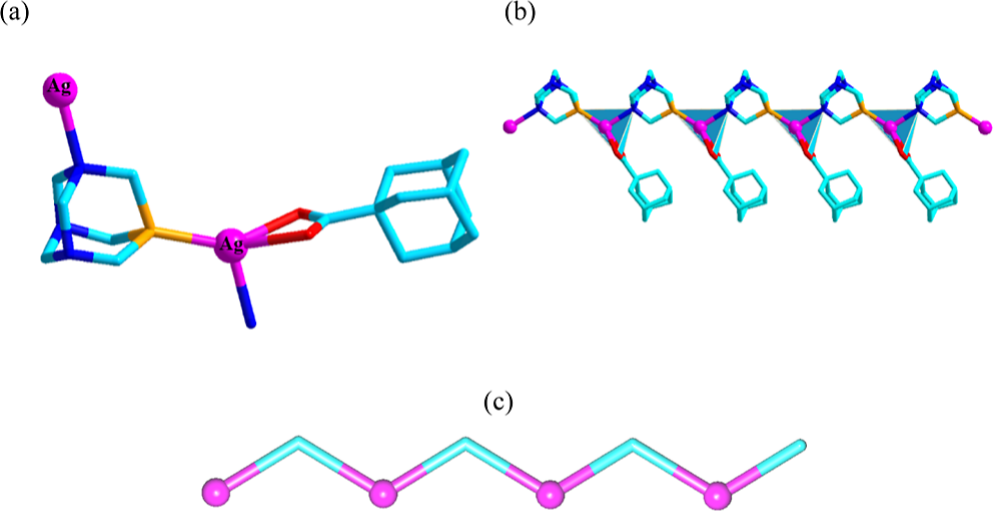
Structural fragments of **1**. (a) Coordination environment of silver center and binding modes of ligands. (b) 1D CP chain and its (c) topological representation. Details: (a,b) H atoms are omitted, Ag (magenta), O (red), C (cyan), N (blue), P (orange); (c) 2C1 topology, Ag centers (magenta), centroids of μ-PTA linkers (cyan).

**Figure 2:**
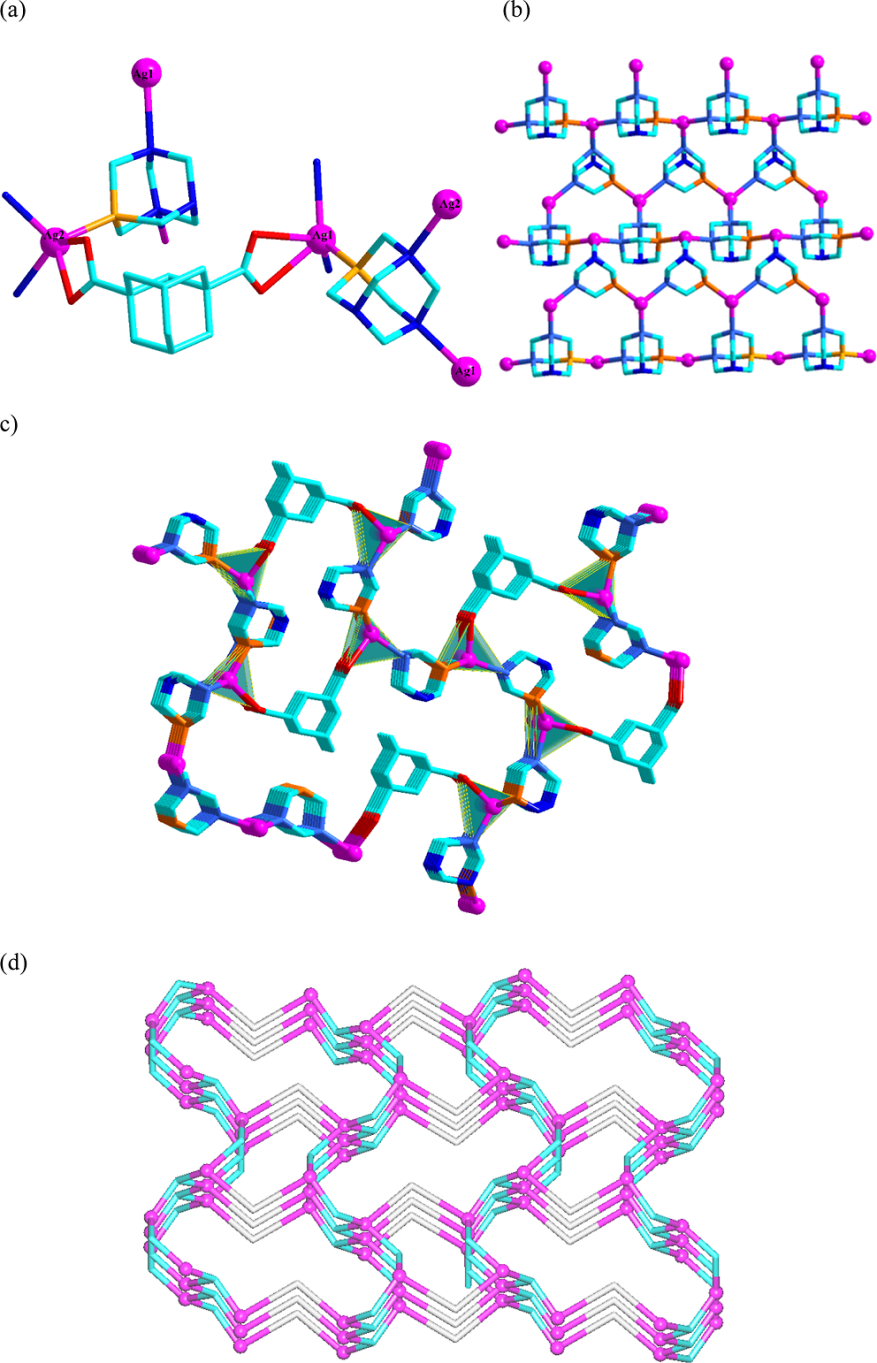
Structural fragments of **2**. (a) Coordination environments of silver centers and binding modes of ligands. (b) 2D Ag-PTA motif. (c) 3D metal–organic network and its (d) topological representation. Details: (a,b) H atoms are omitted, Ag (magenta), O (red), C (cyan), N (blue), P (orange); (c) trinodal 3,4,4-linked net; 4-connected Ag1/Ag2 nodes (magenta), centroids of 3-connected μ_3_-PTA nodes (cyan), centroids of 2-connected μ-adc^2–^ linkers (gray), rotated view along the *b* axis.

The X-ray structures of **1** and **2** feature 1D and 3D metal–organic architectures (Figures 1 and 2). In **1**, the four-coordinate Ag1 atom represents a distorted {AgPNO_2_} environment formed by two carboxylate O atoms, as well as one N_PTA_ and one P_PTA_ donor (Figure 1a). The Ag–P, Ag–O, and Ag–N distances are in the range of 2.339–2.517 Å. The aca^–^ block acts as a chelating terminal ligand, while PTA functions as a P,N-linker. As a result, the Ag1 centers are held into a 1D coordination chain of 2C1 topological type (Figure 1b,c). This chain is driven by the μ-PTA linkers and represents the shortest Ag1...Ag1 separation of 6.887 Å (Figure 1b).

Compound **2** features a significantly more complex 3D metal–organic network structure given the presence of two Ag(I) centers, two μ_3_-PTA blocks, and a μ-ada^2–^ linker (Figure 2). Both Ag atoms are five-coordinated and adopt distorted {AgPN_2_O_2_} environments. These are made of two carboxylate O atoms from μ-ada^2–^ linkers, two N_PTA_ atoms, and one P_PTA_ atom. The tau parameter (τ_5_ = (β – α)/60)^24^ indicates that the geometry is better described as a distorted square pyramidal. The Ag–P, Ag–O, and Ag–N distances are in the range of 2.304–2.621 Å. Each carboxylate group of μ-ada^2–^ exhibits a bidentate chelating mode. The Ag1 and Ag2 centers are bridged by the μ_3_-PTA blocks into wavelike 2D {Ag_2_(PTA)_2_}_*n*_ motifs of a honeycomb type (**hcb** topology, Figure 2b). These 2D layer motifs are further interconnected by both carboxylate groups of the μ-ada^2–^ linkers to form a 3D layer-pillared structure (Figure 2b,c). From a topological viewpoint, this 3D network can be defined as a 3-nodal 3,4,4-linked net with a unique topology and a (6^3^)_2_(6^5^.8)(6^6^) point symbol.

### Synthesis of AHSP and Ag-Doped Biocomposites

**Scheme 1:**
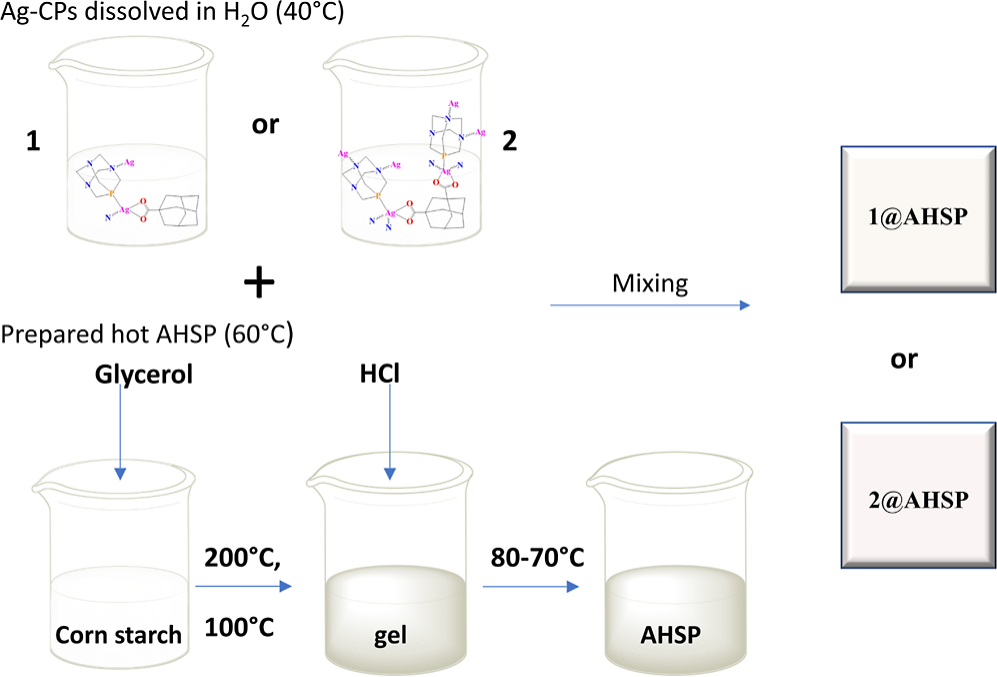
A Simplified Preparation Protocol for **1**@AHSP and **2**@AHSP

The obtained silver(I) CPs were used as dopants (1 wt %) to generate new hybrid biomaterials formulated as **1**@AHSP and **2**@AHSP (Scheme 1). For the preparation of AHSP, we followed a modified synthetic protocol described by Jaimes et al.^13^ In brief, a hot liquid AHSP (60 °C) was combined with an aqueous solution of **1** or **2** and dried in a vacuum dryer, resulting in the formation of hybrid antimicrobial composites **1**@AHSP or **2**@AHSP (Scheme 1). The reaction parameters were optimized. If the temperature is too high when preparing AHSP, silver CPs may start decomposing and the formation of Ag_2_O or Ag(0) nanoparticles can be observed.

The FTIR spectra of the obtained materials do not show a significant difference between pure AHSP and Ag-doped biocomposites (Figures S12–S15). The primary vibrations are observed in the region of C–H stretching bands (2935 to 2880 cm^–1^) and the area from 1155 to 1080 cm^–1^ corresponds to the C–O–C vibrations. Further studies were performed on biofilm squares with a surface area of 9 cm^2^, thickness of 0.40–0.42 mm, and flatness of 100% (Figure S10 and Table S7).

**Figure 3:**
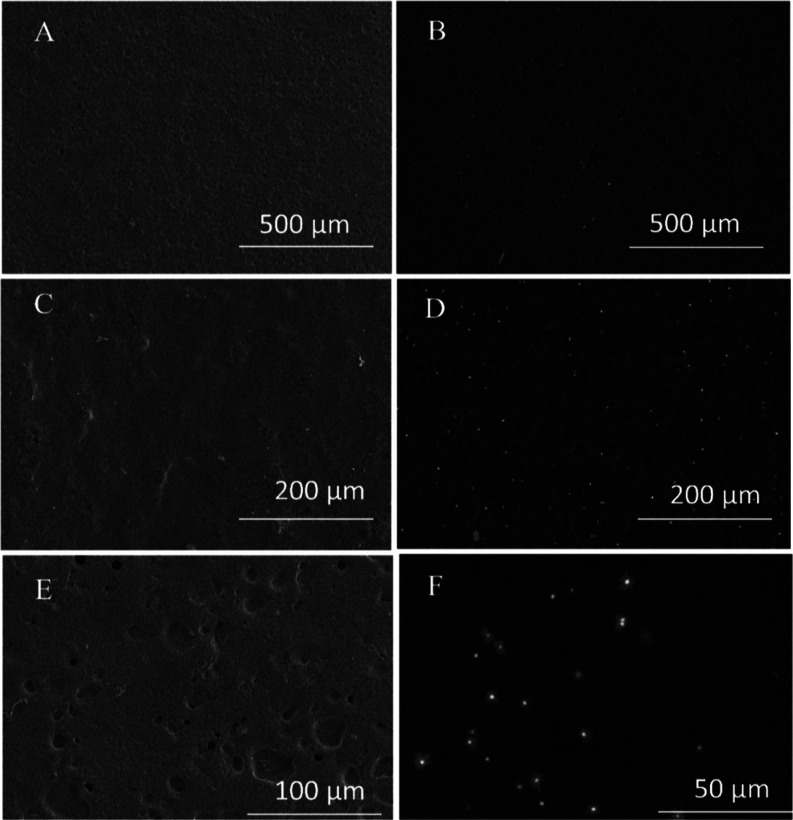
Scanning electron microscopy microphotographs of **1**@AHSP showing the morphology of porous flat surface (A,C) with some visible recesses (E), as well as the distribution of compound **1** (B,D,F).

The morphological characterization of **1**@AHSP and **2**@AHSP by SE–SEM evidences a uniform porous surface or a flat wavy surface, respectively. For **1**@AHSP, we observe evenly distributed pores (Figure 3a) in the film matrix. In the case of **2**@AHSP, the surface is less porous and more uniform (Figure S15a). The composition of materials was determined by the EDS-element mapping, confirming the presence of silver species (Figures S16–S18).

The BSE-SEM (backscattered electron) micrographs also show the presence of Ag species as evidenced by visible bright points coming from high atomic number elements such as Ag that backscatter the electrons stronger than the elements with lower atomic numbers (e.g., P, O, N).^25^ These bright points on micrographs likely come from Ag_2_O formed during the aging upon the storage of **1**@AHSP and **2**@AHSP at room temperature. Electrospray ionization-mass spectrometry (ESI-MS) measurements of the **1**@AHSP and **2**@AHSP samples confirmed that the materials contain different complex species, and we suggest that these fragments are responsible for biological activity. We observed that both materials start to brown over time, which may indicate the formation of Ag_2_O or Ag particles. However, even after one year, neither material was completely brown or black. Based on the distribution of these bright points we can conclude about the distribution of **1** and **2** in **1**@AHSP and **2**@AHSP. **1**@AHSP, we observe evenly distributed pores (Figure 3A,C,E) in the biofilm matrix. Although the Ag^+^ species are nearly homogeneously distributed in **1**@AHSP, some local concentrations of **1** on the surface can be observed (Figure 3B,D,F) in the BSE-SEM micrographs. In the case of **2**@AHSP, the surface is less porous and more uniform (Figure S15a). Compared to **1**@AHSP, the Ag^+^ ions are less uniformly distributed on the surface of **2**@AHSP (Figures S16b–d).

Both **1**@AHSP (a) and **2**@AHSP exhibit a moderate uptake of moisture and the ability to absorb water from air. Moreover, the ICP-OES measurement confirmed that the release of Ag^+^ ions from **1**@AHSP and **2**@AHSP is time dependent. Both materials show a resembling behavior (Figure S11), with an increase in the Ag^+^ concentration from 0.06 nmol/mL (after 4 h) to 0.12–0.21 nmol/mL (after 20 h). The latter values correspond to a release of 0.37 and 0.39% from all silver present in **1**@AHSP and **2**@AHSP, respectively.

### ESI-MS Measurements

The behavior of solutions containing **1** and **2** as well as the samples obtained after soaking **1**@AHSP and **2**@AHSP in water was investigated in detail by ESI/MS spectrometry (including high-resolution mass spectrometry and collision-induced dissociations (CID) measurements of isolated isotopic charged species). The experiments were repeated twice on materials from different syntheses. In the case of water medium (solutions of **1** and **2**), the high-resolution full scan mass spectra obtained in the 100–1000 *m*/*z* range displayed a set of characteristic cationic species for both compounds, namely *m*/*z* 263.9798, 421.0589, 707.0650 (for **1**), and 751.0548 (for **2**) assigned to [Ag(PTA)]^+^, [Ag(PTA)_2_]^+^, [Ag_2_(PTA)_2_(aca)]^+^, and [Ag_2_(PTA)_2_(Hada)]^+^, respectively. The low-resolution mass spectra acquired in the 100–2000 *m*/*z* range also showed sets of cationic clusters of **1** and **2** attributed to *m*/*z* 993 [Ag_3_(PTA)_2_(aca)_2_]^+^, 1150 [Ag_3_(PTA)_3_(aca)_2_]^+^, 1436 [Ag_4_(PTA)_3_(aca)_3_]^+^, 1722 [Ag_5_(PTA)_3_(aca)_4_]^+^, or 1014 [Ag_3_(PTA)_3_(ada)]^+^ and 1501 [Ag_4_(PTA)_4_(ada)(Hada)]^+^, respectively (Figures S7 and S8). These results indicate that the stock solutions of both compounds contain the resembling type of species. This assumption is supported by the MS^2^ spectra obtained by CID of selected precursor ions, which follow identical dissociation pathways. The main fragmentation processes observed in the MS^2^ spectra of precursor ions with *m*/*z* 707 and 751 are due to the direct loss of 157 mass units (a PTA molecule), leading to the [Ag_2_(PTA)(ada)]^+^ (*m*/*z* 550 for **1**) and [Ag_2_(PTA)(Hada)]^+^ (*m*/*z* 594 for **2**) species. There is also the elimination of the [Ag(Hacid)] moiety, resulting in the most stable [Ag(PTA)_2_]^+^ ion at *m*/*z* 421, as illustrated for **2** (Figure S9). The latter is the most abundant species in all the MS^2^ spectra, as well as the base peak in all the MS spectra for **1** and **2**. These results confirm that despite different dimensionality and structural features, the carboxylate silver(I)-PTA derivatives after electrospray ionization display similar product ions and fragmentation patterns in the solution.^22−24^

Further ESI/MS experiments assessed the solution composition after soaking the biocomposites **1**@AHSP and **2**@AHSP in an aqueous medium (10 mL). One hour after immersion of both biocomposites in H_2_O at room temperature, aliquots of the media were collected, and their chemical composition was determined by high-resolution mass spectrometry. The full mass spectra of both bulk solutions displayed a base peak at *m*/*z* 421.0586 attributed to a [Ag(C_6_H_12_N_6_P)_2_]^+^ ion, which follows an expected fragmentation.^22−24,26^ These spectra also present a very low-intensity peak centered at *m*/*z* 562.9321. Its observed isotopic distribution pattern agrees with the one calculated for [Ag_2_(C_6_H_12_N_6_P)_2_Cl]^+^ (Figure S19). The formation of this cation is likely related to the synthetic procedure of AHSP that involves the use of a small volume of HCl solution.

### Antiviral Properties

The obtained CPs and hybrid biopolymers were tested for their potential virucidal properties (Table 1) against HSV-1 and human adenovirus (HAdV-5). We observed that the reduction rates of both HSV-1 and HAdV-5 for **1** were 4 log (99.99% reduction). Compound **2** showed a similar reduction rate against HAdV-5 and a slightly lower 3.5 log reduction against HSV-1. This activity is comparable with acyclovir (4 log reduction of HSV-1 titer) which is, however, inactive against HAdV-5. The free ligands Haca and H_2_ada show significantly lower virucidal activity against HSV-1, namely 2.5 and 3.5 log reduction, respectively. In the case of HAdV-5, the 2.5 and 3 log reductions are observed for Haca and H_2_ada, respectively.

**Table 1:** Comparison of the Virucidal Properties of the Tested Compounds and Biocomposite Materials (Expressed in the Logarithmic Scale and % Reduction)

virus	**1**	**2**	**1**@AHSP	**2**@AHSP	acyclovir
**HSV-1**	4 log (99.99%)	3.5 log (99.99%)	4 log (99.99%)	1.66 log (96.6%)	4 log (99.99%)
**HAdV-5**	4 log (99.95%)	4 log (99.99%)	1.33 log (93.3%)	1.66 log (96.6%)	n.d^a^

a Not determined.

Importantly, the biofilms doped with a low amount of **1** and **2** also exhibit a pronounced virucidal behavior. In fact, **1**@AHSP features the same 4 log reduction of HSV-1 and a 1.33 log reduction (93.3% reduction) of HAdV-5 titers. The **2**@AHSP material exhibits a 1.66 log reduction (96.6% reduction) against both tested viruses.

The exact mechanism of action of CPs **1** and **2** is not yet known. Based on the studies conducted with amantadine (also known as 1-aminoadamantane hydrochloride) on other viruses, it is likely to involve the blockage of viral protein ion channels.^27−31^ Amantadine can be considered as structurally related to adamantanoid ligands in compounds **1** and **2** and has been an approved antiviral drug since 1966 for type A influenza prevention and treatment. The antiviral action of this tricyclic adamantane-derived amine is related to the interaction with M2 channels. Many viruses contain small hydrophobic proteins with ion channel activity known as viroporins. They are small, usually less than 100 amino acids in length with transmembrane domains (1 or 2 TMDs).^27^ One of the viroporins is the M2 protein of the influenza A virus. It serves also as a target for amantadine and rimantadine. Probably, M2 has a proton translocation function capable of regulating the pH of vesicles of the trans-Golgi network, a role important in promoting the correct maturation of the hemagglutinin glycoprotein.^28^

Another known target for amantadine is viroporin p7 of HCV (hepatitis C virus). This is confirmed by the fact that an L20F mutation in this protein confers resistance to this drug in combined therapy with IFN-α.^31−35^ Several viroporins were recognized in some amantadine-sensitive flaviviruses. In the case of DENV (Dengue virus), there are the 2K peptide, the membrane protein (M), and the nonstructural proteins NS2A and NS2B. For WNV (West Nile virus), the M protein serves as a viroporin.^31^

Brown et al. used in vitro rimantadine and demonstrated a reduction in the expression of ZIKV (Zika virus) envelope protein (E). The potential role of viroporin activity in ZIKV infection is also played by the M protein. Additionally, in vivo ZIKV preclinical models were used to confirm that rimantadine reduces viremia, supporting that M protein channel activity is a relevant physiological target to block ZIKV infection.^31^ The mechanism of adamantine-mediated blockade of HSV-1 and HAdV-5 replication has not been described so far. However, the high virucidal activity of **1** and **2** against both viruses makes them excellent candidates for antiviral use.

It is noteworthy that **1** shows full efficacy against the enveloped HSV-1, both as a solution and as a dopant to a biocomposite material. This compound also significantly reduces the levels of nonenveloped adenovirus. Hence, it might become a promising surface material in many products exposed to viral contamination and further investigated as a topical herpes treatment as a patch applied to lesions caused by the virus.

### Antibacterial and Antifungal Activity

Both silver(I) CPs were designed to have a broad spectrum of antimicrobial activity. The minimum inhibitory concentrations (MICs) were investigated using the serial dilution method.^24^ Compounds **1** and **2** displayed a marked but varying antibacterial and antifungal activity (Table 2). Both substances were most efficient against the gram-negative bacteria [except *Klebsiella pneumoniae* (*K. pneumoniae*)], for which the MIC values ranged from 7 to 8 μg mL^–1^. Gram-positive bacteria [*S. aureus*, *Bacillus cereus* (*B. cereus*)] and the gram-negative species *K. pneumoniae* turned out to be more resistant, with MIC values of 20 μg mL^–1^ for both compounds. The least activity of CPs **1** and **2** (20–40 μg mL^–1^) was observed against the yeasts *Cryptococcus neoformans* (*C. neoformans*) and *C. albicans*. Interestingly, *C. neoformans* (which is a difficult-to-treat pathogen) displayed susceptibility similar to that of gram-positive bacteria rather than to yeast. Compared to the action of AgNO_3_ (a well-recognized topical antibacterial), compounds **1** and **2** showed equal or slightly higher antimicrobial activity. However, if the MIC values are normalized for the molar content of silver, compounds **1** and **2** are far more active (except *B. cereus* and *K. pneumoniae*) in comparison with the silver(I) nitrate reference. The normalized values also revealed some differences in the activities of **1** and **2**. The PTA, Haca, and H_2_ada ligands alone were not active against the tested microorganisms even at the highest concentration screened (60 μg mL^–1^). The observed disparity in antimicrobial activities against particular groups of microorganisms may result from different compositions of the cell envelopes. Gram-negative bacteria have a relatively thin cell wall, so they could be more susceptible to silver ions. Gram-positive bacteria, on the other hand, are likely to absorb a larger amount of silver ions to their multiple layers of peptidoglycan in the cell wall, thus being more resistant.^32^ Similarly, in the case of other microorganisms with thick external structures (e.g., thick cell walls in fungi or heavy capsules in *K. pneumoniae*), the activity of silver(I) compounds was distinctly weaker.

**Table 2:** MICs of **1** and **2** against Bacteria and Yeasts Tested in the Present Study

Microorganism	MIC [μg mL^–1^]^a^			Normalized MIC^b^ [nmol mL^–1^]		
	**1**	**2**	AgNO_3_	**1**	**2**	AgNO_3_
Gram-Negative Bacteria						
*E. coli*	8	7 (6–8)	9	17	18	53
*P. aeruginosa*	7(6–8)	7 (6–8)	9	15	18	53
*Bordetella bronchiseptica*	7 (6–8)	7 (6–8)	<1	15	18	<18
*K. pneumoniae*	20	20	4 (3–5)	42	52	24
Gram-Positive Bacteria						
*S. aureus*	20	20	20	42	52	118
*B. cereus*	20	20	5	42	52	29
Yeasts						
*C. albicans*	40 (20–60)	40 (30–50)	40	83	104	236
*C. neoformans*	20	20	n.t^c^	42	52	n.t

a Expressed as a mean of six replications (if results were discrepant, the ranges obtained were given in parentheses).

b Values normalized for a molar content of silver in the compounds.

c Not tested.

### Surface Activity of Ag-Doped Starch Biopolymers against Microorganisms

The surface activity of the obtained materials against bacteria and yeasts was tested by the application of normalized microbial suspensions in tryptic soy broth onto square-shaped starch polymers (blank and doped with compounds **1** or **2**; Figures 1, S10, and S23). Microorganisms were next washed off the squares and transferred on a growth medium to count living cells (CFUs, Table 3). The activity was analyzed after the contact time 0 and 24 h (T0 and T24, respectively). The microorganisms tested, depending on the species, displayed varying abilities to multiply on blank starch squares. However, in no case, a reduction in the number of microorganisms was detected. After 24 h of incubation (T24), **2**@AHSP caused complete inhibition of all the microbial strains tested. In the case of **1**@AHSP, the same was observed for *E. coli* and *S. aureus*, whereas growth of *P. aeruginosa* and *C. albicans* was only partially suppressed (by 3 log and less than 1 log, respectively). The positive control test with AgNO_3_@AHSP could not be performed for comparison as this material decomposes very quickly due to the reduction of silver ions.

**Table 3:** Surface Activity of **1**@AHSP and **2**@AHSP against Microorganisms^a^

species	AHSP (control)		**1**@AHSP (T24) **2**@AHSP (T24)	
	T0	T24	no. of CFU (log reduction)	
*E. coli*	1.0 × 10^6^	1.8 × 10^8^	0 (6)	0 (6)
*S. aureus*	1.8 × 10^6^	2.2 × 10^9^	0 (6.25)	0 (6.25)
*P. aeruginosa*	4.8 × 10^6^	9.0 × 10^6^	4.8 × 10^3^ (3)	0 (9.68)
*C. albicans*	1.6 × 10^5^	4.4 × 10^5^	9.4 × 10^4^ (0.3)	0 (5.18)

a The data show the mean number of CFU of bacteria and yeast detached from the biopolymer squares after 24 h of contact as well as the calculated logarithm of viable cell reduction.

Because the adamantane moiety has a lipophilic character, its incorporation into various bioactive molecules results in compounds with a relatively high lipophilicity, which modifies the bioavailability and modulates the therapeutic efficacy.^32−40^ Nowadays, the adamantane-like fragments are often introduced into the structures of otherwise active drugs to improve pharmacological properties,^33,35^ or to enhance the stability and distribution of the drug in blood plasma.^36,37^ Several adamantane derivatives have been recognized as potent antibacterial and/or antifungal agents.^38−53^ For example, SQ109 (a 1,2-ethylenediamine derivative originated from ethambutol),^44^ which is approved for the use against drug-susceptible and drug-resistant *Mycobacterium tuberculosis* strains, also shows an excellent inhibitory activity against *Candida glabrata*.^47−51^ Another example concerns adamanta-platensimycin, a bioactive analog of platensimycin (a newly discovered antibiotic, isolated from *Streptomyces platensis*, exhibiting potent activity against gram-positive bacteria).^49^ While analyzing the antimicrobial activity of various adamantyl derivatives, other research groups noticed different patterns of susceptibility of particular bacteria. Most of the compounds examined^23,50−58^ were more active against the gram-positive bacteria than gram-negative ones. However, in the experiments employing silver(I) derivatives^23^ as well as in the present study, Gram-negative bacteria were more susceptible. These results indicate that various compounds containing adamantoid motifs, irrespective of their primary biological properties and antimicrobial activity, can be additionally tailored to better address the intended therapeutic or preventive purpose.

In the present study, two silver derivatives of 1,3,5-triaza-7-phosphaadamantane and adamantanecarboxylic acids (CPs **1** and **2**) showed evident antimicrobial activity, both in solution and as dopants to starch polymers, thus proving their contact antimicrobial action. The antimicrobial activity of these two new compounds and the respective biomaterials can be related to the increasing penetration of metal species through the bacterial membrane (in particular, the lipidic membrane) as a result of enhanced lipophilicity. Based on the silver ion release tests, the concentration of silver ions increases with time. From **1**@AHSP and **2**@AHS immersed in PBS, there is a release of 0.04 and 0.06 nmol/mL of Ag^+^ ions within 4 h, respectively. After 20 h, the Ag^+^ concentration attains 0.22 and 0.12 nmol/mL for **1**@AHSP and **2**@AHSP, respectively. This low concentration is enough to ultimately reduce the number of tested pathogenic species in the case of **2**@ASHP. In contrast, the obtained concentration of Ag^+^ from **1**@ASHP is not sufficient to completely reduce *P. aeruginosa* and *C. albicans* strains. The HR mass spectrometry tests indicate similar solution behavior of both materials after being submerged in water with the release of stable [Ag(PTA)_2_]^+^ cations and not free silver ions. The solution speciation is similar for both compounds. However, [Ag(PTA)_2_]^+^ ion concentration and release speed differ (Figure S11). Moreover, we observed the formation of [Ag_2_(PTA)_2_Cl]^+^ species in the presence of a trace amount of Cl^–^. The formation of this ion cannot be excluded in culture media and physiological conditions and may exert a significant role in the mechanism of antimicrobial action of these types of compounds. However, more detailed studies are required to elucidate the mechanism of antimicrobial action of these silver(I) adamantane derivatives.

### Antioxidant Properties

Antioxidant behavior of **1** and **2** was investigated in the peroxidation of LinMe (methyl linoleate) in the micellar system (Triton X-100) at pH 7.0 (the plots of oxygen uptake are shown in Figure S24). Autoxidation was initiated by the addition of 0.5 M (final concentration 10 mM) of water-soluble azo-initiator, 2,2′-azobis(2-amidinopropane) dihydrochloride (AAPH). We monitored the rates of peroxidation in the presence of both compounds **1** and **2**, and the ligand precursors Haca and H_2_ada (Figure S24). The kinetic plots are substantially the same compared to AAPH and only slight changes in line slopes are observed. Such results indicate that some of the investigated compounds might be weak retardants of the peroxidation of LinMe in the micellar system. Among the tested compounds, **2** is kinetically neutral (contrary to the ligand from which it was made). Based on these results, we can conclude that the complexation of H_2_ada changes its antioxidant activity. However, the antioxidant properties of Haca are the same before and after the complexation to **1** (both the compounds **1** and free ligand exhibit similar behavior). In the case of both ligands and **1**, we observe a slight inhibition of the autoxidation without visible induction time. This might be due to a localization problem as these compounds are likely localized in the water phase and neutralize the primary alkyl radicals before they enter the micelles. Although the observed antioxidant behavior of ligands and **1** is not very effective, they are not kinetically neutral during the peroxidation of micelles initiated with AAPH. Hence, these compounds could behave as retardants. Undoubtedly, tested compounds are not pro-oxidants.

### Interaction of **1** and **2** with Human Serum Albumin

The rational design of a new therapeutic compound requires a basic understanding of its interaction with the blood component. In particular, identifying the nature of interactions of bioactive compounds and transported proteins in blood is essential for therapeutic application. Serum albumin is the most prevalent protein in human blood and, due to its binding properties, acts as a carrier of a wide range of endogenous and exogenous molecules. Consequently, we investigated the interaction of **1** and **2** with HSA using fluorescent spectroscopy to elucidate the structural elements that influence their biological activity.^59−61^ This technique was used to determine the quenching mechanism of HSA fluorescence by **1** and **2** and to calculate the binding and thermodynamic parameters of the systems. Generally, two types of quenching are observed: static and dynamic. Static quenching occurs when a complex between fluorophore and quencher is observed, whereas dynamic quenching is caused by collision. Titration experiments with **1** and **2** revealed a slight quenching of the protein fluorescence and a blueshift of the emission peak by 2 nm (for details, see Supporting Information). This behavior indicates the interaction between human serum albumin and metal compounds and some conformational changes in the protein. The maximum scatter collision quenching constant indicates the contribution of the static quenching mechanism of the protein fluorescence by the compounds. This refers to the formation of HSA-**1** or HSA-**2** complexes (for details, see Supporting Information).^61^ The *n* values of approximately 1 in both systems indicate that only one binding site in protein is accessible for the complex. The binding constants of the systems are in the order of 10^2^–10^3^ M^–1^. These are comparable with the value of HSA–cisplatin (*K*_a_ = 8.52 × 10^2^ M^–1^).^62^ For comparison, the association constants of the ruthenium complex tested in clinical trials KP1339 are *K*_a_ = 4.57 × 10^4^ M^–1^.^63^ The negative value of calculated Δ*H*^0^ and Δ*S*^0^ indicates that the van der Waals interactions and hydrogen bonds are the leading forces in the HSA-**1** and HSA-**2** systems (Table S1).

Circular dichroism was used to study the conformational change in HSA resulting from any binding. Human serum albumin is mostly an α-helical protein (Table S2), indicating no significant changes in its structure. The interaction with **1** and **2** remotely affects the secondary structure of the protein, breaking up its hydrogen bonding network. Nevertheless, an α-helical structure is still a dominant form of the protein.

### Docking Studies

To assess the ability of fragmented CP networks to interact with viral proteins, docking studies were carried out. Two proteins were selected for this part: the M2 proton channel of influenza A virus (PDB code 6BKK)^67^ and the p7 cationic channel of the hepatitis C virus (PDB code 2M6X).^64^ The former structure contains an amantadine fragment, which was used as a control marker to assess the docking accuracy. It is hypothesized that the antiviral action of the amantadine class of drugs is related to water-lined sites of the host protein that stabilize transient oxonium ions formed in the proton-conduction mechanism.^65^

**Figure 4:**
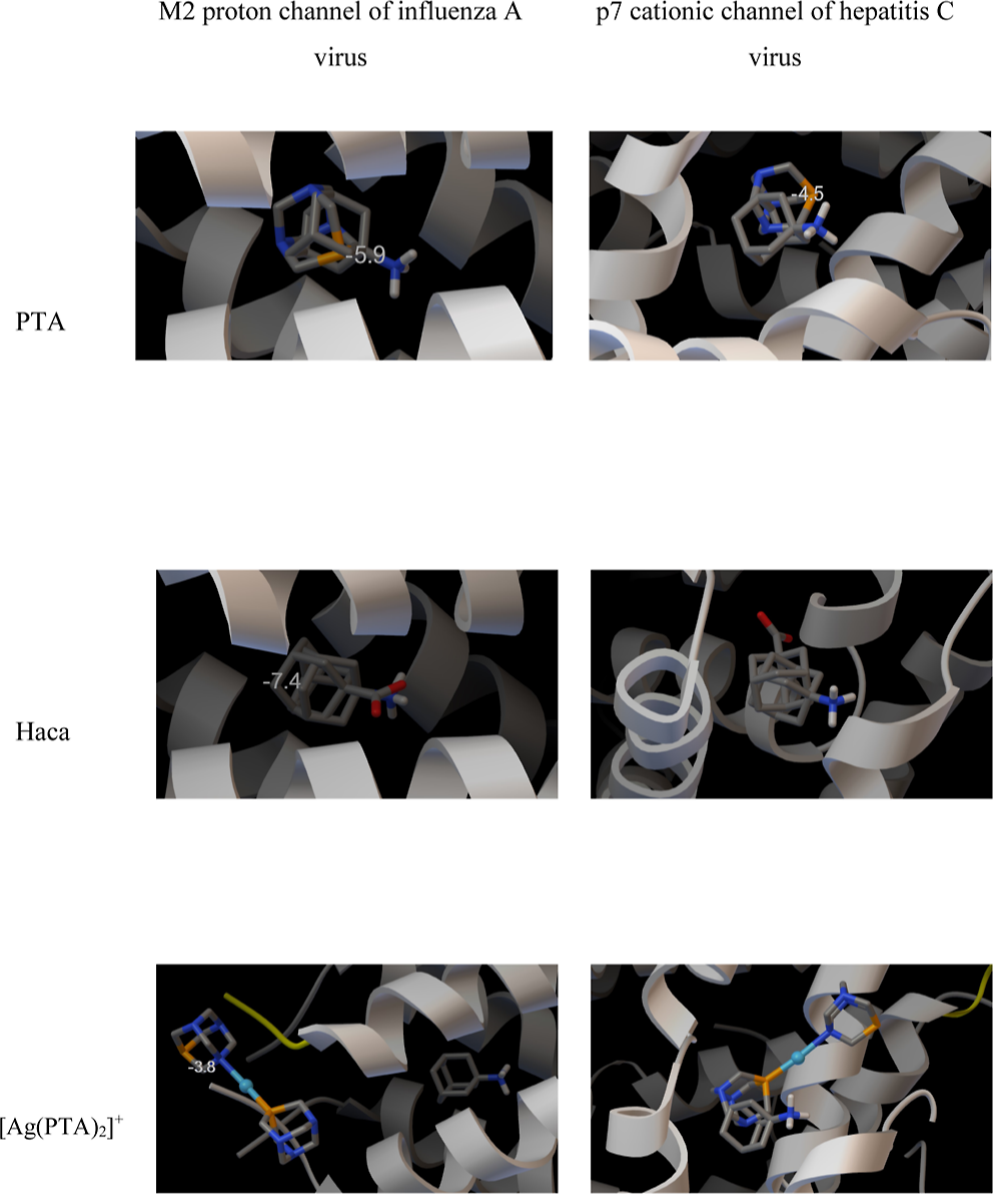
Docking results for PTA, Haca, and [Ag(PTA)_2_]^+^ as ligands and two channel proteins as hosts, respectively. The protein structures were taken from the following PDB deposits: 6BKK for the influenza A channel, and 2M6X for the hepatitis C virus. Positions of amantadine (experimental for 6BKK and docked for 2M6X) are shown as a control marker to assess the docking accuracy.

The only structures for which successful docking results were obtained were the free ligands (PTA, Haca) and the [Ag(PTA)_2_]^+^ fragment. The structures of these species docked into the two host proteins are depicted in Figure 4, while the corresponding binding affinities are grouped in Table 4. The location of amantadine (experimental for 6BKK and docked for 2M6X) is included in Figure 4. Docking of free ligands is easily possible for the M2 influenza A protein, where strong overlap occurs between the experimental marker (amantadine) and the docked molecules. The binding affinity of PTA (−5.9 kcal/mol) is lower than that of the prototypic amantadine (−7.0 kcal/mol) but, on the other hand, Haca binds even more strongly (−7.4 kcal/mol). However, the [Ag(PTA)_2_]^+^ species is too large and could not be successfully docked, because its binding affinity is smaller if compared to free ligands and the docked pose is well outside of the central pore of the channel. This would suggest that at least in part the biological action of the investigated CPs is related to the bioactive ligands present in their structures.

**Table 4:** Binding Affinities [kcal/mol] of Amantadine (AMT, Used as a Control), PTA, Haca, and [Ag(PTA)_2_]^+^^a^

	M2 proton channel of influenza A virus (6BKK)	p7 cationic channel of hepatitis C virus (2M6X)
AMT (control)	–7.0	–5.4
PTA	–5.9	–4.5
Haca	–7.4	–6.5
[Ag(PTA)_2_]^+^	–3.8	–6.9

a The target molecules are viral membrane cationic channels.

The situation is different for the cationic channel of hepatitis C virus p7 that possesses a much larger central pore. Maximum binding efficiency is found for [Ag(PTA)_2_]^+^ and, in all cases, there is a good match between the positions of amantadine and the docked ligands. The binding affinities calculated within the docking force field approximation are rather semiquantitative; therefore, it is possible to conclude that in this case, the smaller fragments of the polymer present in the aqueous solution can be responsible for the biological activity.

We would like to underline that, even considering the docking force field as only semiquantitative, the diverse species present in the dissolved CP can exert antiviral action according to the mechanism reported for the amantadine class of drugs.

## Conclusions

In this study, we described a facile and straightforward synthesis of two new silver(I) compounds, namely 1D [Ag(aca)(μ-PTA)]_*n*_·5*n*H_2_O (**1**) and 3D [Ag_2_(μ-ada)(μ_3_-PTA)_2_]_*n*_·4*n*H_2_O (**2**) CPs, which are based on two different adamantane-like building blocks. These compounds were fully characterized and used as bioactive Ag(I) dopants to obtain hybrid starch-based biocomposite films **1**@AHSP and **2**@AHSP. Both the obtained Ag(I) CPs and derived biopolymer materials revealed an exceptional virucidal activity against HSV-1 and HAdV-5 viruses with up to 4 log virus titer reduction (99.99% reduction). Besides, these materials disclosed a remarkable antibacterial and antifungal activity with regard to clinically relevant microbial species.

The anionic adamantane-like ligands in compounds **1** and **2** are responsible for their polymeric architecture and stabilization of silver ions. The collective effect of different Ag–N, Ag–O, and Ag–P bonding thus resulted in a promising platform for creating multifunctional Ag^+^-ion-releasing materials. These incorporate adamantoid building blocks that, despite not being antibacterial, show recognized antiviral properties. Hence, the combination of anionic carboxylate ligands with antimicrobial silver ions and neutral PTA with partial water-solubility is an interesting strategy toward multifunctional and bioactive CPs, as well as hybrid starch-based materials incorporating CPs as dopants. The broad spectrum of antimicrobial activity and, particularly, antiviral activity of the obtained hybrid materials resulted from the synergistic action of all used components.

Considering the antioxidant measurements, the compounds **1** and **2** do not act as pro-oxidants. Compound **2** is kinetically neutral, whereas **1** exhibits a weak retardation activity. Depending on the concentration, retardants are able to slow down the rate of reaction but cannot stop it completely. Both compounds demonstrated a relatively weak affinity to human serum albumin, forming unstable adducts under physiological conditions. Moreover, protein conformation does not change significantly, during the interaction with **1** and **2**. In the course of such an interaction, HSA retains its structure and function, which is important from the pharmacological point of view. Docking of molecular fragments of **1** and **2** to the influenza and hepatitis virus channel proteins reveals the binding affinities that are similar to the antiviral drug, amantadine.

Based on such results, we hypothesize that the potent antiviral, antibacterial, and antifungal activity of the tested compounds is not a result of their pro-oxidant properties. Moreover, the high contact antimicrobial potential of Ag-doped AHSP materials and, in particular, **2**@AHSP may have significance in the development of antimicrobial surfaces and materials that can reduce viral, bacterial, and fungal contamination. The research on further exploration of these bioactive silver(I) CPs and derived biopolymer materials and interfaces is currently in progress.

## Experimental Section

### [Ag(aca)(μ-PTA)]_*n*_·5*n*H_2_O (**1**)

Silver(I) oxide (0.1 mmol, 23 mg), 1-adamantanecarboxylic acid (Haca; 0.25 mmol, 45.1 mg), and PTA (0.25 mmol, 31 mg) were combined in a solution containing MeOH (7 mL) and H_2_O (3 mL) and stirred in air for 1 h. The produced white-colored suspension was dissolved by a dropwise addition of NH_4_OH (until pH = 8; ∼ 0.8 mL, 1 M in H_2_O). The resulting solution was filtered off and the filtrate was left in a vial to slowly evaporate in air at room temperature, leading to the formation of colorless crystals in 2 days. These were collected, washed with H_2_O and CH_3_OH, and dried in air to give **1** in 60% yield, based on Ag_2_O. *S*_25 °C_ in H_2_O: 4 mg·mL^–1^. Elemental analysis: C_17_H_29_AgN_3_O_3_P (**1** + H_2_O): (MW 462.3): C, 44.17; N, 9.09; H, 6.32. Found: C, 44.48; N, 8.91; H, 5.52. IR (KBr, cm^–1^): 3436 (s br) ν(H_2_O + OH), 2902 (s) ν_as_(CH), 2849 (m), 1627 (w), 1547 (s) ν_as_(COO), 1452 (w), 1404 (m) ν_s_(COO), 1363 (w), 1343 (w), 1293 (m), 1307 (w), 1287 (m), 1241 (s), 1180 (w), 1100 (m), 1040 (w), 1014 (vs), 975 (vs), 950 (w), 900 (w), 797 (m), 752 (m), 720 (w), 678 (w), 603 (m), 577 (w), 565 (w), 511 (m), 478 (w), 449 (w). ^1^H NMR (300 MHz, D_2_O): δ 4.64 and 4.53 (2d, 6H, *J*_AB_ = 13.73 Hz, NC*H*^A^*H*^B^N, PTA), 4.27 (s, 6H, PCH_2_N, PTA) 1.93 (s, 3H, aca), 1.78 (s, 6H, aca), 1.69 and 1.66 (2d, 6H, aca). ^31^P{^1^H} NMR (202.5 MHz, D_2_O): δ −77,93 (s, PTA). ESI-MS(+) (H_2_O), MS(+) *m*/*z* (relative abundance,%): 158 (30%) [Ag(H_2_O)]^+^, 421 (90%) [Ag(PTA)_2_]^+^, 709 (100%) [Ag_2_(PTA)_2_(aca)]^+^, 1153 (40%) [Ag_3_(PTA)_3_(aca)_2_]^+^, 1439 (10%) [Ag_4_(PTA)_3_(aca)_3_]^+^.

### [Ag_2_(μ-ada)(μ_3_-PTA)_2_]_*n*_·4*n*H_2_O (**2**)

Compound **2** was synthesized via a procedure described for **1** but using 1,3-adamantanedicarboxylic acid (H_2_ada; 0.25 mmol, 56.1 mg) instead of Haca. Colorless crystals of **2** were isolated in 40% yield, based on Ag_2_O. *S*_25 °C_ in H_2_O: 2 mg mL^–1^. Elemental analysis: C_24_H_58_Ag_2_N_6_O_14_P_2_ (**2** + 10H_2_O): MW 752.3: C, 30.91; N, 9.01; H, 6.27. Found: C, 31.11; N, 8.88; H, 6.30. IR (KBr, cm^–1^): 3421 (s br) ν(H_2_O + OH), 2933 (m) ν_as_(CH), 2854 (w), 1641 (w) and 1542 (vs) ν_as_(COO), 1438 (w), 1390 (vs) ν_s_(COO), 1312 (w), 1286 (vs), 1236 (s), 1123 (w), 1099 (w), 1093 (m), 1040 (w), 1013 (vs), 964 (vs), 952 (m), 898 (m), 881 (w), 807 (w), 794 (m), 722 (w), 750 (m), 727 (w), 677 (w), 596 (s), 585 (s), 564 (w), 450 (m), 399 (m). ^1^H NMR (300 MHz, D_2_O): 4.59 and 4.48 (2d, 12H, *J*_AB_ = 12.97 Hz, NC*H*^A^*H*^B^N, PTA), 4.23 (d, 6H, PCH_2_N, J = 2.3 PTA) 2.03 (m, 3H, ada), 1.75 (s, 2H, aca), 1.73, 1.70 and 1.67 (m, 9H, ada). ^31^P{^1^H} NMR (202.5 MHz, D_2_O): δ −83,60 (s, PTA). ESI-MS(+) (H_2_O), MS(+) *m*/*z* (relative abundance,%): 158 (15%) [Ag(H_2_O)]^+^, 421 (100%) [Ag(PTA)_2_]^+^, 753 (95%) [Ag_2_(PTA)_2_(Hada)]^+^, 1016 [Ag_3_(PTA)_3_(ada)]^+^.

### Synthesis of **1**@AHSP and **2**@AHSP

AHSP was prepared by adapting the described protocol^27^ and using glycerol as a plasticizer. Corn starch (2.5 g), (Sigma-Aldrich) was mixed with distilled water (25 mL) and glycerol (1% w/w). The obtained white mixture was stirred using a mechanical stirrer and fast heated in an oil bath (200 °C) until it began to boil. Then, the temperature was reduced to 100 °C and the reaction mixture was kept heating for 10 min to produce a colorless gel. After cooling down to ca. 80–70 °C and gelatinization, HCl (0.1 M in H_2_O, 120 μL) was added and the mixture was stirred for 30 min. The pH was monitored while washing AHSP several times with boiling water (10 mL) until achieving a neutral pH. Then, hot AHSP (60 °C) was combined with a hot (40 °C) solution of **1** and **2** in water (15 mL) in quantities necessary to obtain the **1**-1%@AHSP and **2**-1%@AHSP doped biofilms. The hot dense mixture was then stirred for 10 min on a mechanical stirrer and poured onto hot sterilized Petri dishes (9 mm, kept for 1 h at 180 °C). The obtained films of **1**@AHSP and **2**@AHSP were dried in the drying chamber at 30 °C for 2 to 4 h (or in air at room temperature for 3 to 4 days). **1**@AHSP. IR (KBr, cm^–1^): 3429 (vs br) ν(H_2_O + OH), 2932 (m ν_as_(CH), 1636 (m), 1419 (w), 1312 (w), 1155 (w), 1110 (w), 1080 (w), 1041 (m), 925 (m), 860 (m), 574 (w). **2**@AHSP. IR (KBr, cm^–1^): 3419 (vs br) ν(H_2_O + OH), 2935 (m) ν_as_(CH), 2151 (w), 1644 (s), 1417 (w), 1206 (w), 1155 (w), 1110 (w), 1080 (w), 1041 (m), 925 (m), 859 (m), 670 (w), 574 (w).

### X-ray Crystallography

Single crystal data collection was performed on an Xcalibur diffractometer (Oxford Diffraction) with Sapphire2 CCD detector, equipped with an Oxford Cryosystems open-flow nitrogen cryostat, using ω-scan and a graphite-monochromated Mo K_α_ (λ = 0.71073 Å) radiation. Cell refinement, data reduction, analysis, and absorption correction were carried out with CrysAlis PRO (Rigaku Oxford Diffraction) software.^66^ Both structures were solved by direct methods by means of SHELXT-2014/5 and refined against *F*^2^ using the SHELX-2019/2 program.^67^ The selected bond lengths and angles are listed in Tables S1–S6. The crystal structure of **1** was initially solved and refined up to *R*_1_ = 0.1052 and the maximum difference peak of 6.235 e Å^–3^ (*R*_int_ = 0.0363). TwinRotMat function of the PLATON program^68,69^ determined the twin law (rotation around [201] axis) and generated a two-component HKLF5 file, leading to *R*_1_ = 0.0860 and the highest difference peak of 4.409 e Å^–3^ and BASF parameter of 0.225. Two pairs of atoms (N1/P1 and N3/P2) of the PTA ligands in **2** were found to be positionally disordered with site occupancies of 0.875 (N1A, P1A, N3A, and P2A) and 0.125 (N1B, P1B, N3B, and P2B), where the atoms in the pairs N1A/P1B, P1A/N1B, N3A/P2B, and P2A/N3B share the same sites. The hydrogen atoms of water molecules in **1** and **2** were localized and refined with O–H and H···H intramolecular separations restrained to 0.96 and 1.53 Å, respectively, and *U*_iso_ = 1.5*U*_eq_ of the parent oxygen atoms. The remaining H atoms in **1** and **2** were placed at calculated positions and refined using the riding model with *U*_iso_ = 1.2*U*_eq_. CCDC 2222401 (**1**) and 2222402 (**2**).

Crystal data for **1**: C_17_H_37_AgN_3_O_7_P (M = 534.33 g mol^–1^), monoclinic, space group *P*2_1_/*c*, *a* = 24.3875(8) Å, *b* = 6.8182(3) Å, *c* = 13.7706(4) Å, β = 105.664(4)°, *V* = 2204.72(14) Å^3^, *Z* = 4, *T* = 100(2) K, μ = 8.396 mm^–1^, *D*_calc_ = 1.610 g cm^–3^, 15,398 reflections measured (3.765° ≤ Θ ≤ 71.819°) 4194 unique, 3921 with *I* > 2σ(*I*), *R*_1_ = 0.0860 (*I* > 2σ(*I*)), w*R*_2_ = 0.2897 (all data).

Crystal data for **2**: C_24_H_46_Ag_2_N_6_O_8_P_2_ (M = 824.35 g mol^–1^), orthorhombic, space group *Pnma*, *a* = 17.1973(3) Å, *b* = 6.46570(10) Å, *c* = 27.0741(3) Å, *V* = 3010.44(8) Å^3^, *Z* = 4, *T* = 100(2) K, μ = 11.934 mm^–1^, *D*_calc_ = 1.819 g cm^–3^, 37,070 reflections measured (3.044° ≤ Θ ≤ 76.181°), 3384 unique, 3216 with *I* > 2σ(*I*), *R*_1_ = 0.0492 (*I* > 2σ(*I*)), w*R*_2_ = 0.1293 (all data).

## Supporting Information Available

Crystallographic data for **1** (CIF)
Crystallographic data for **2** (CIF)
Materials and methods; additional experimental data including IR, NMR, and ESI-MS spectra; PXRD and TGA plots; and additional results (PDF)

## Supplementary Material

am3c15606_si_001.cif

am3c15606_si_002.cif
