# Supplementary material for: Silver Coordination Polymers Driven by Adamantoid Blocks for Advanced Antiviral and Antibacterial Biomaterials
Source: ACS Appl Mater Interfaces. 2024 Mar 8;16(11):13411–21. doi: 10.1021/acsami.3c15606 (PMC10958451; doi:10.1021/acsami.3c15606)
Supplement: Supplementary file 3 — am3c15606_si_003.pdf [file am3c15606_si_003.pdf]

Supporting Information (SI)

Silver Coordination Polymers Driven by Adamantoid Blocks for Advanced Antiviral and Antibacterial Biomaterials

Sabina W. Jaros,[†]* Magdalena Florek,[‡] Barbara Bażanów,[‡] Jarosław Panek,[†] Agnieszka Krogul-Sobczak,[§] M. Conceição Oliveira,[#] Jarosław Król,[‡] Urszula Śliwińska-Hill,[⊥] Dmytro S. Nesterov,[#] Alexander M. Kirillov,[#]* Piotr Smoleński[†]

[†] Faculty of Chemistry, University of Wrocław, F. Joliot-Curie 14, 50-383 Wrocław, Poland; sabina.jaros@uwr.edu.pl

[‡] Department of Veterinary Microbiology, Wrocław University of Environmental and Life Sciences, Norwida 31, 50 -375 Wrocław, Poland;

[§] Faculty of Chemistry, University of Warsaw, Pasteura 1, 02-093 Warsaw, Poland;

[#] Centro de Química Estrutural, Institute of Molecular Sciences, Departamento de Engenharia Química, Instituto Superior Técnico, Universidade de Lisboa, Av. Rovisco Pais, 1049–001 Lisbon, Portugal; kirillov@tecnico.ulisboa.pt

[⊥] Faculty of Pharmacy, Department of Basic Chemical Sciences, Wrocław Medical University, Borowska 211, 50-566 Wrocław, Poland

Contents

Supporting Figures

S1. PXRD patterns of **1** S-4

S2. PXRD patterns of **2** S-4

S3. FTIR spectrum of **1** S-5

S4. FTIR spectrum of **2** S-5

S5. ^{1}H NMR spectrum of **1** S-6

S6. ^{1}H NMR spectrum of **2** S-6

S7. Low resolution full scan mass spectrum acquired in the ESI positive mode for **1** S-7

S8. Low resolution full scan mass spectrum acquired in the ESI positive mode for **2** S-7

S9. MS^2 mass spectrum obtained in the ESI positive mode of the precursor ion $[Ag_2(PTA)_2(Hada)]^+$ *m/z* 751 for **2** S-8

S10. Photographic representation of thickness values of biopolymer films S-8

S11. Silver ion release profile in psychological-like conditions of biofilms **1**@AHSP and **2**@AHSP S-9

S12. FTIR spectrum of the stock corn starch S-9

S13. FTIR spectrum of AHSP S-10

S14. FTIR spectrum of **1**@AHSP S-10

S15. FTIR spectrum of **2**@AHSP S-11

S16. SEM images of **2**@AHSP S-12

S17. EDS-element mapping of **2**@AHSP S-12

S18. EDS-element mapping of **1**@AHSP S-13

S19. HR full scan mass spectra obtained in the ESI positive mode for solutions after immersing **1**@AHSP and **2**@AHSP in water S-13

S20. TGA plot of the starting AHSP S-14

S21. TGA plot of **1**@AHSP S-15

S22. TGA plot of **2**@AHSP S-15

S23. Images of AHSP samples used for antimicrobial assays S-16

S24. Oxygen uptake for autoxidation of micellar system initiated with AAPH S-17

S25. Stern-Volmer plots of the **1**–HSA and **2**–HSA systems S-17

S26. Double logarithm regression plots for quenching of HSA by **1** and **2** S-18

S27. Effect of **1** and **2** concentrations on CD spectra of HSA S-18

Materials and Methods

M1. Materials, Reagents and General Methods S-19
M2. Thickness and Flatness Measurements S-20
M3. Cell Lines and Media S-20
M4. Antiviral Assays S-20
M5. Antimicrobial Activity S-21
M6. Surface Activity of Starch Polymer Doped by **1** and **2** S-22
M7. Autoxidation Measurements S-23
M8. Silver release and stability studies S-24
M9. Interaction of **1** and **2** with Human Serum Albumin S-23
M10. Computational Studies S-26
M11. Moisture Uptake S-27

Supporting Tables

Table S1. Binding and thermodynamic parameters for the interaction of HSA with the studied compounds at different temperatures S-25
Table S2. The percentage of HSA secondary structure in the absence and presence of **1** and **2** calculated with the CDPro software S-26
Table S3. Selected bond lengths for **1** S-28
Table S4. Selected bond angles for **1** S-28
Table S5. Hydrogen bonding parameters for **1** S-28
Table S6. Selected bond lengths for **2** S-29
Table S7. Selected bond angles for **2** S-29
Table S8. Hydrogen bonding parameters for **2** S-30
Table S9. Physicochemical parameters of tested biomaterials S-31

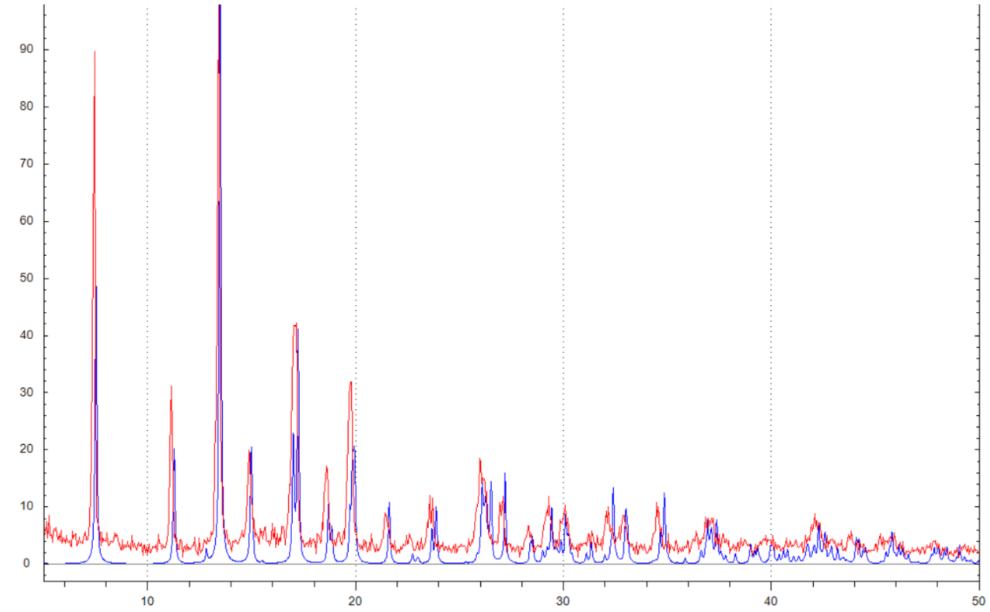

Figure S1. PXRD patterns of compound **1**: calculated from the single-crystal X-ray data (blue), experimental for bulk product (red).

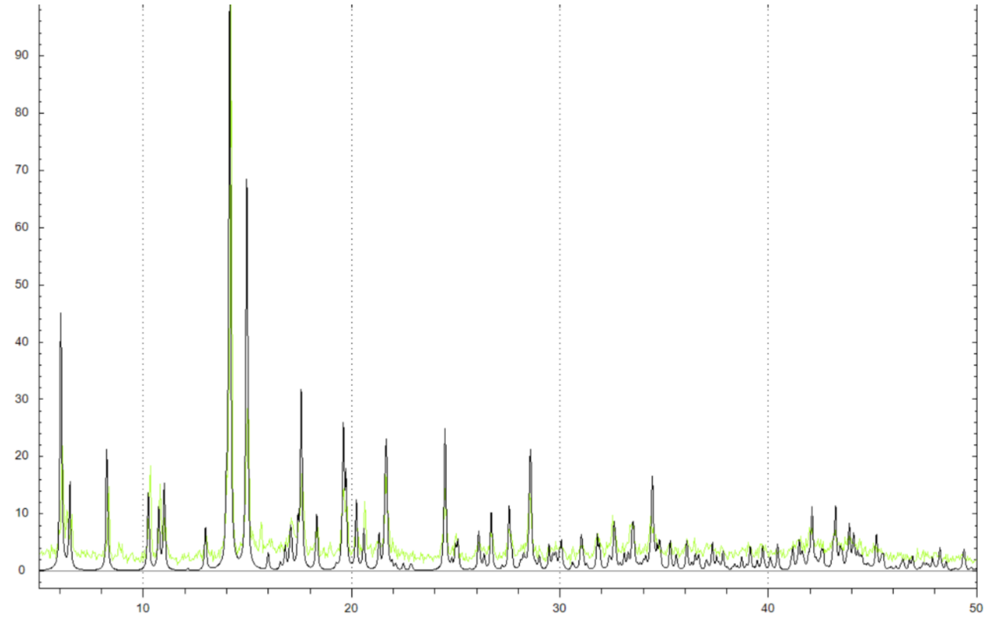

Figure S2. PXRD patterns of compound **2**: calculated from the single-crystal X-ray data (blue), experimental for bulk product (red).

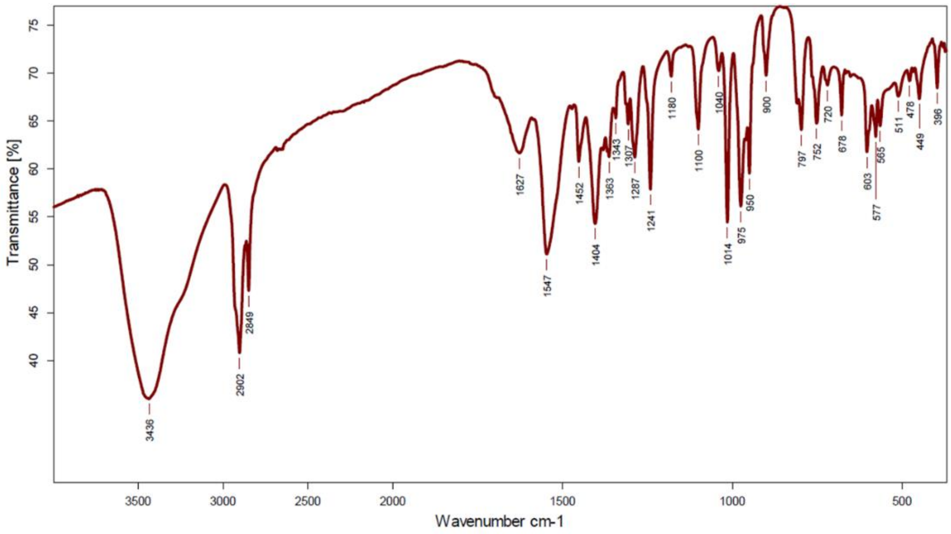

Figure S3. FT-IR spectrum of compound **1**.

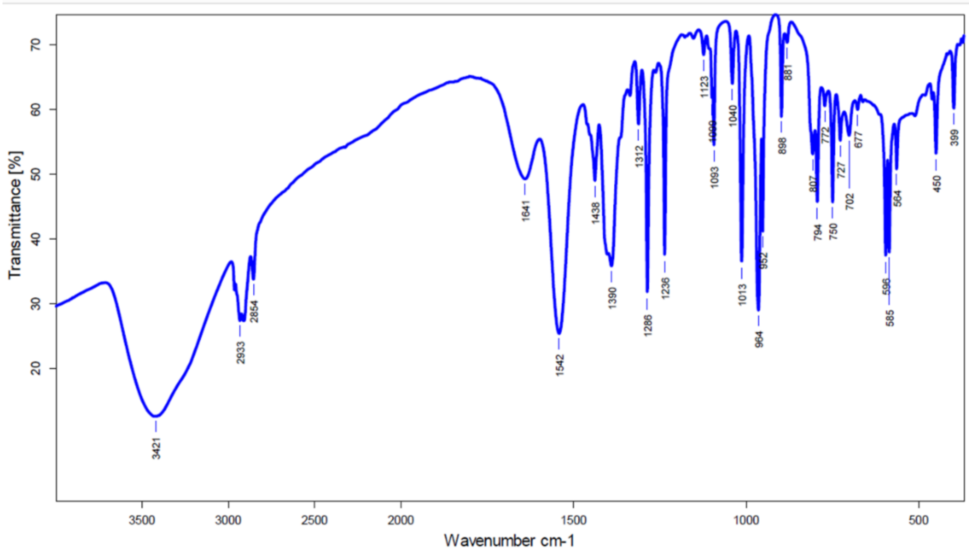

Figure S4. FTIR spectrum of compound **2**.

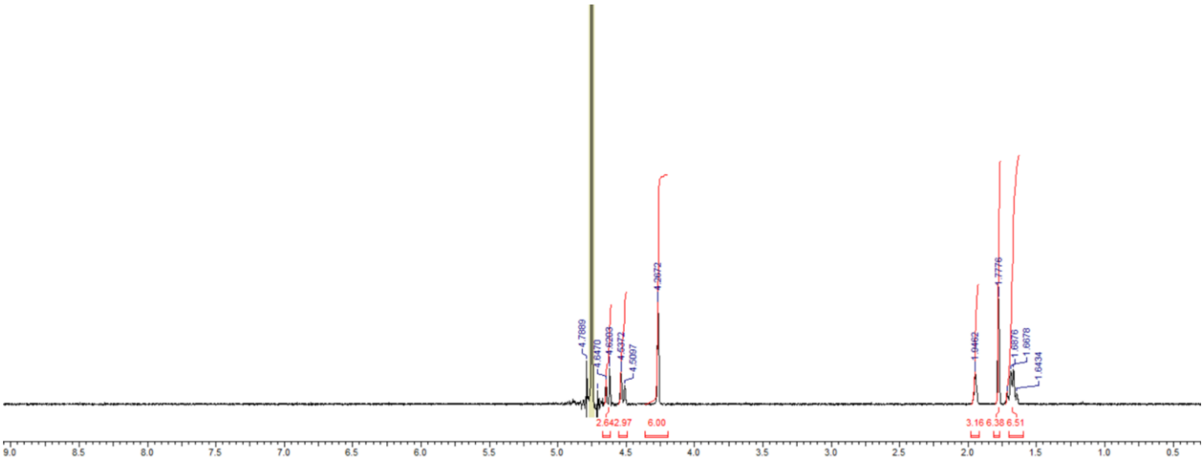

Figure S5. ^{1}H NMR spectrum of **1** in D_2O.

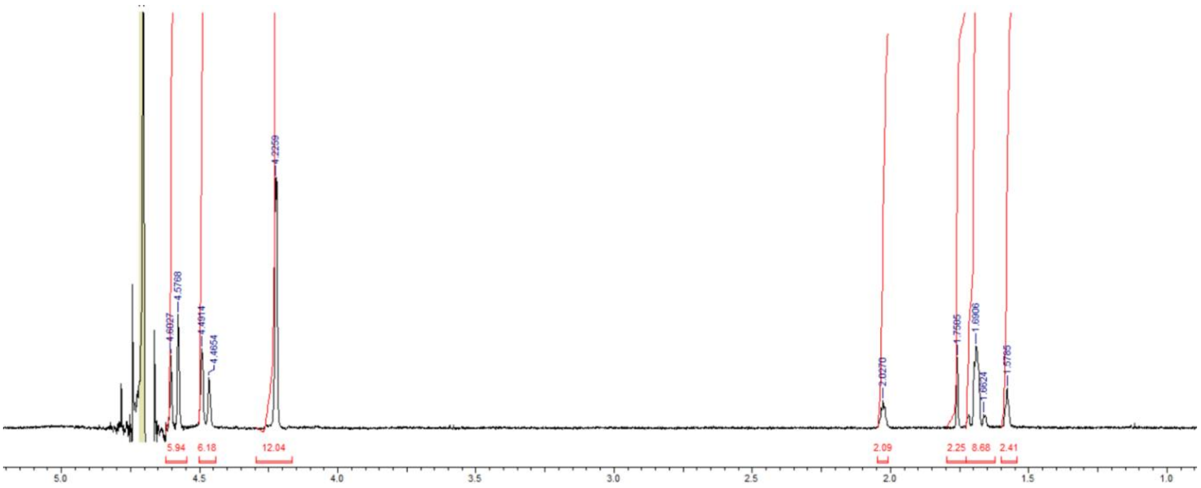

Figure S6. ^{1}H NMR spectrum of **2** in D_2O.

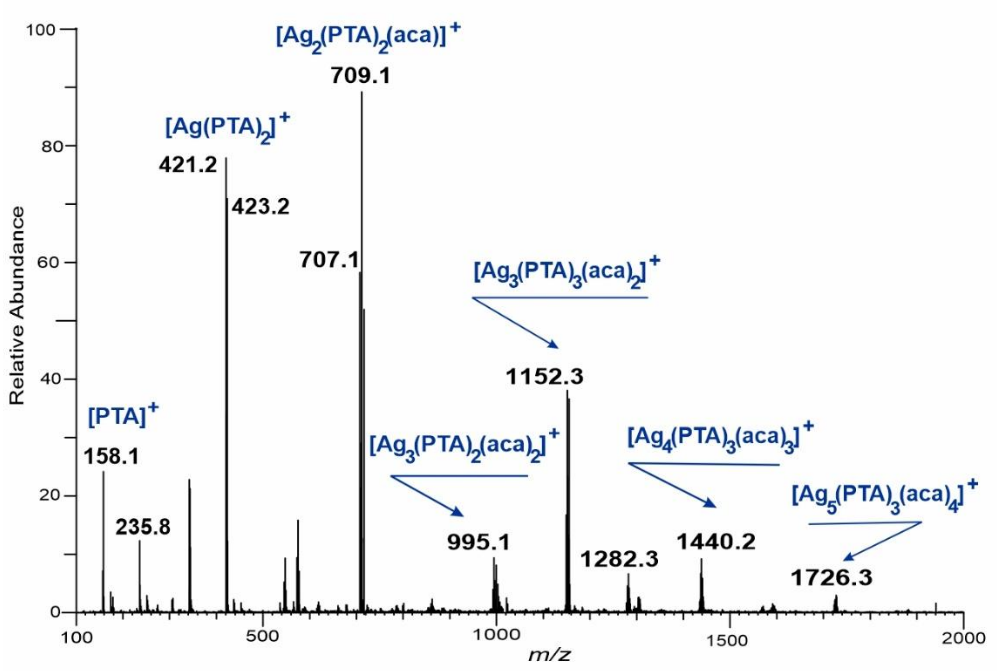

Figure S7. Low resolution full scan mass spectrum acquired in the ESI positive mode for **1** in aqueous medium.

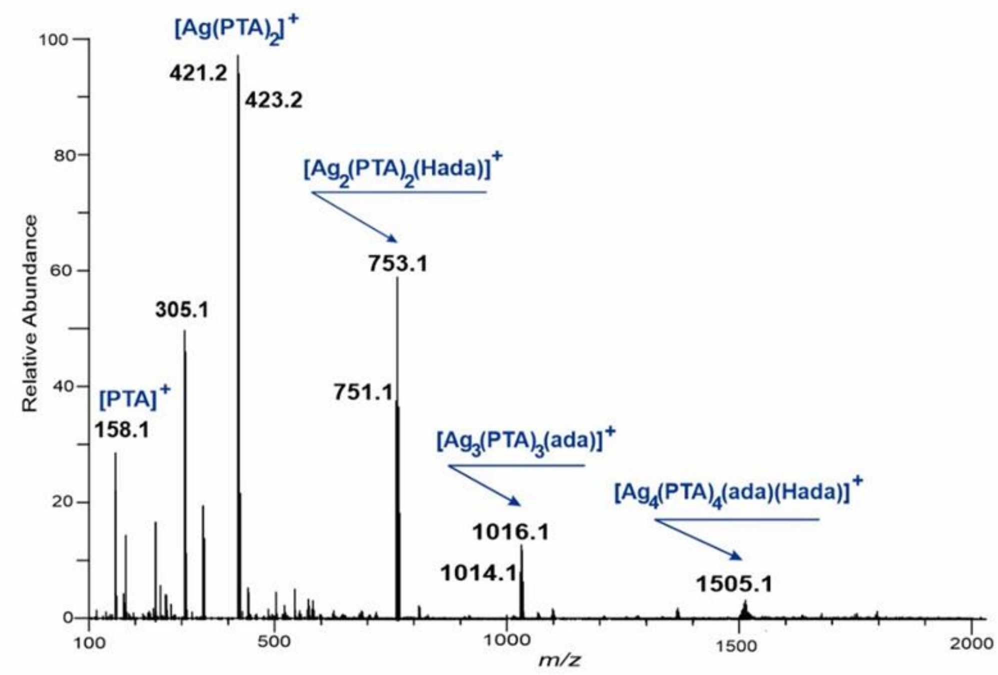

Figure S8. Low resolution full scan mass spectrum acquired in the ESI positive mode for **2** in aqueous medium.

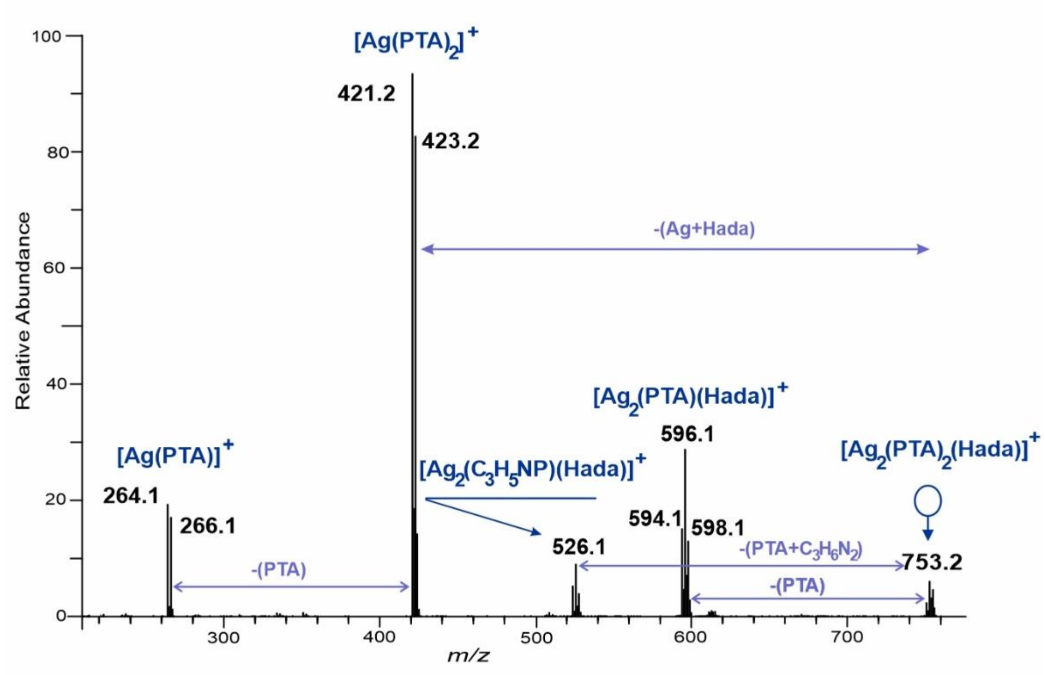

Figure S9. MS^2 mass spectrum obtained in the ESI positive mode of the precursor ion $[Ag_2(PTA)_2(Hada)]^+$ *m/z* 751 for **2** in aqueous medium.

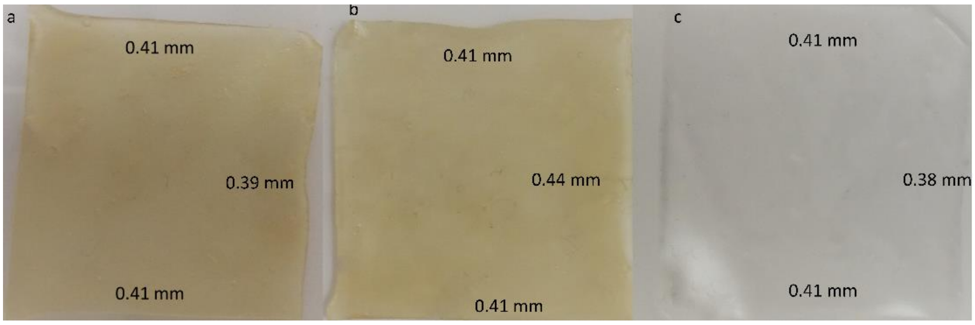

Figure S10. Photographic representation of thickness values of biopolymer films: (a) **1**@AHSP, (b) **2**@AHSP, and (c) AHSP.

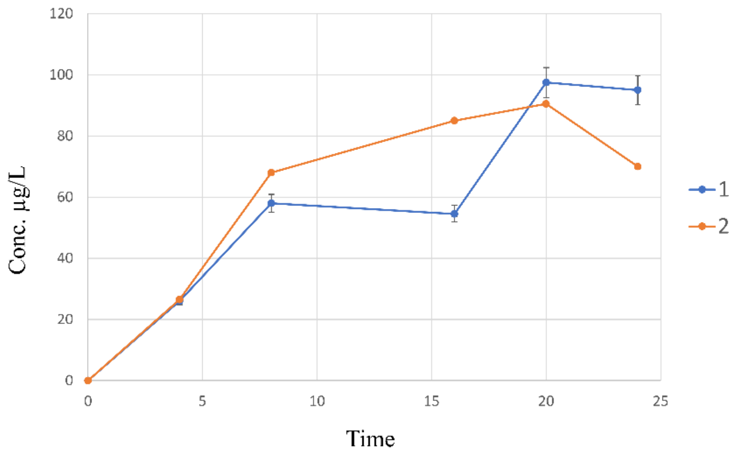

Figure S11. Silver ion release profile in psychological-like conditions from **1**@AHSP (1) and **2**@AHSP (2). For each test, six samples were collected in four-hour intervals.

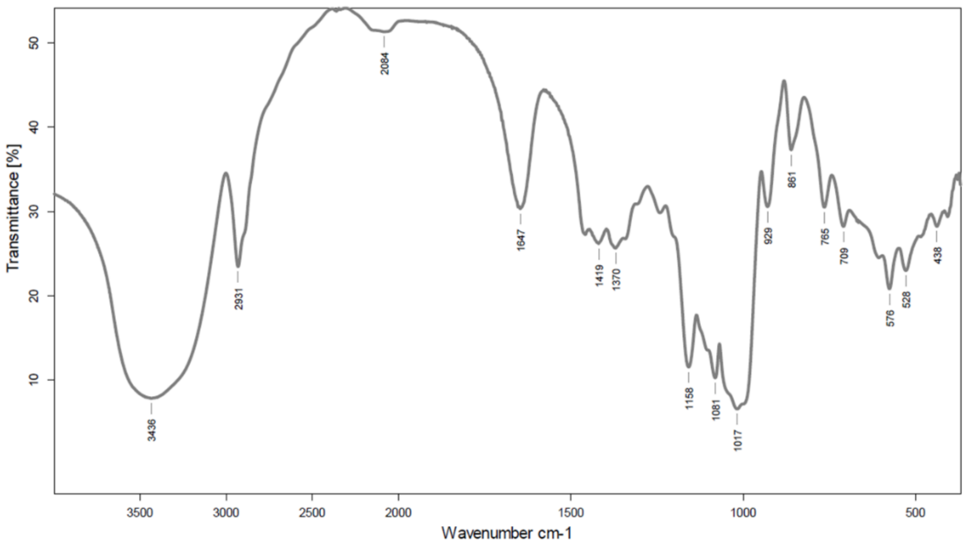

Figure S12. FTIR spectrum of the stock corn starch.

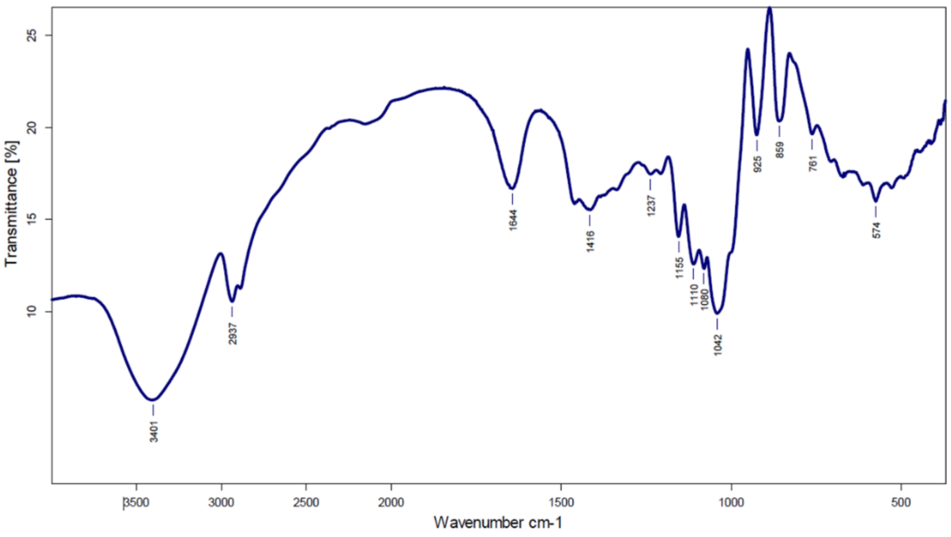

Figure S13. FTIR spectrum of AHSP.

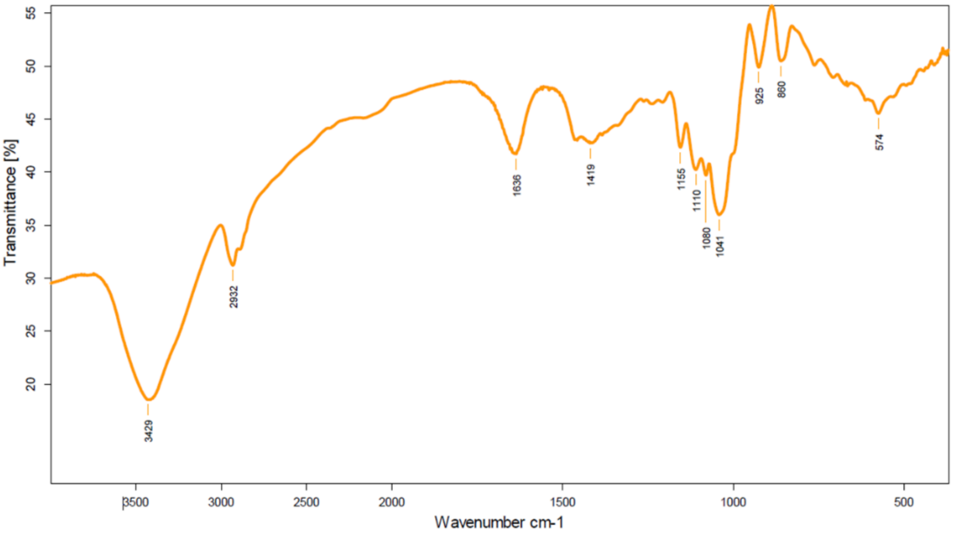

Figure S14. FTIR spectrum of **1**@AHSP.

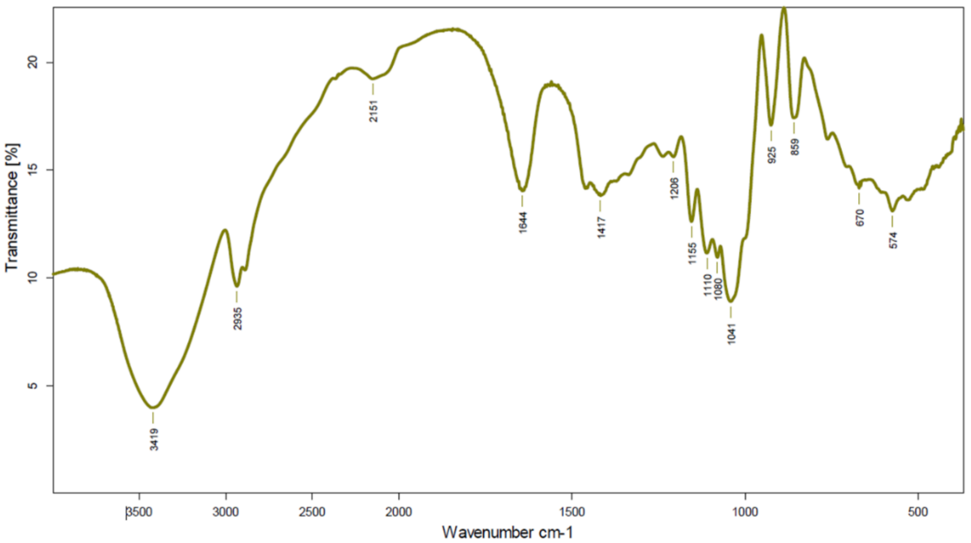

Figure S15. FTIR spectrum of **2**@AHSP.

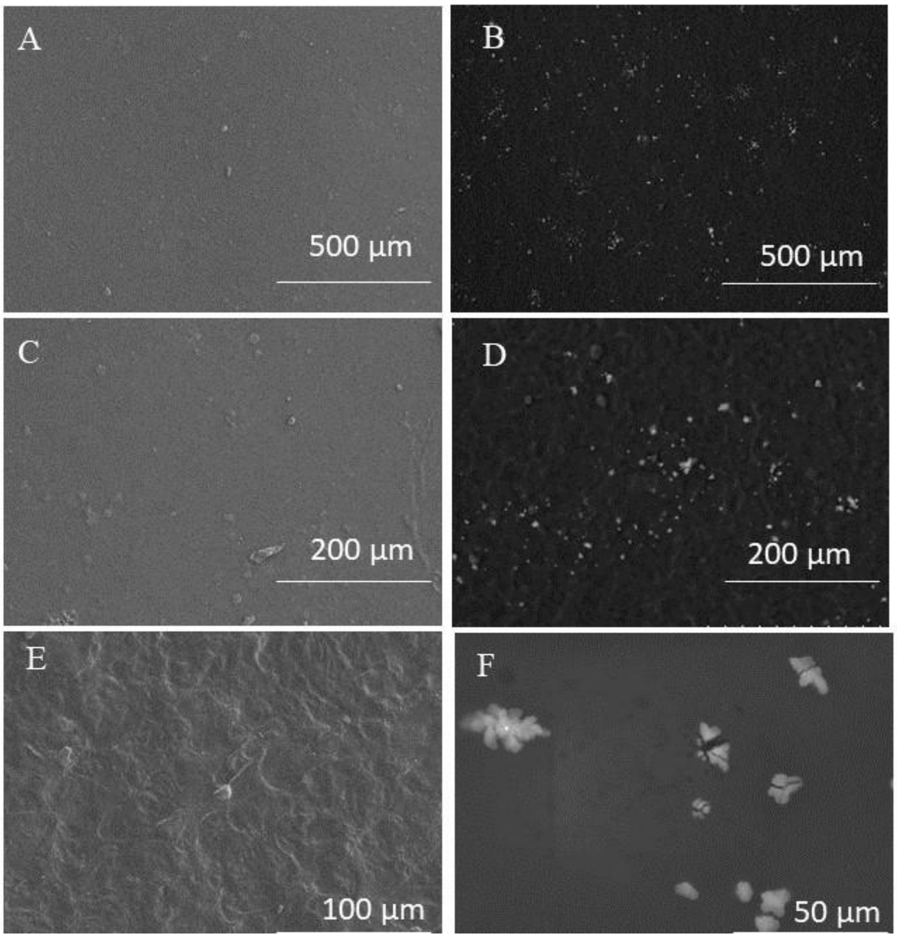

Figure S16. SEM images of **2**@AHSP showing the morphology of porous flat surface (a,c) with some visible recesses (e) as well as distribution of compound **2** (b,d,f).

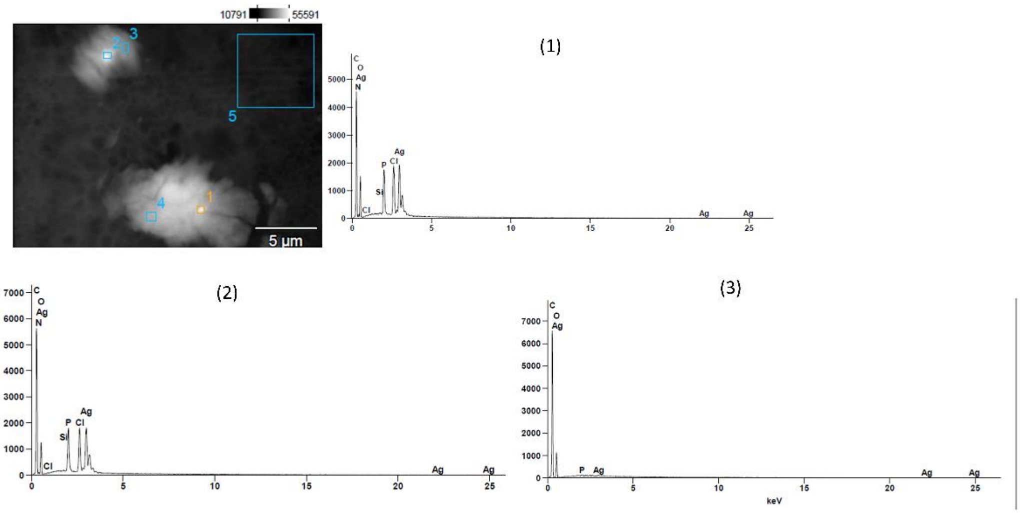

Figure S17. EDS-element mapping of **2**@AHSP.

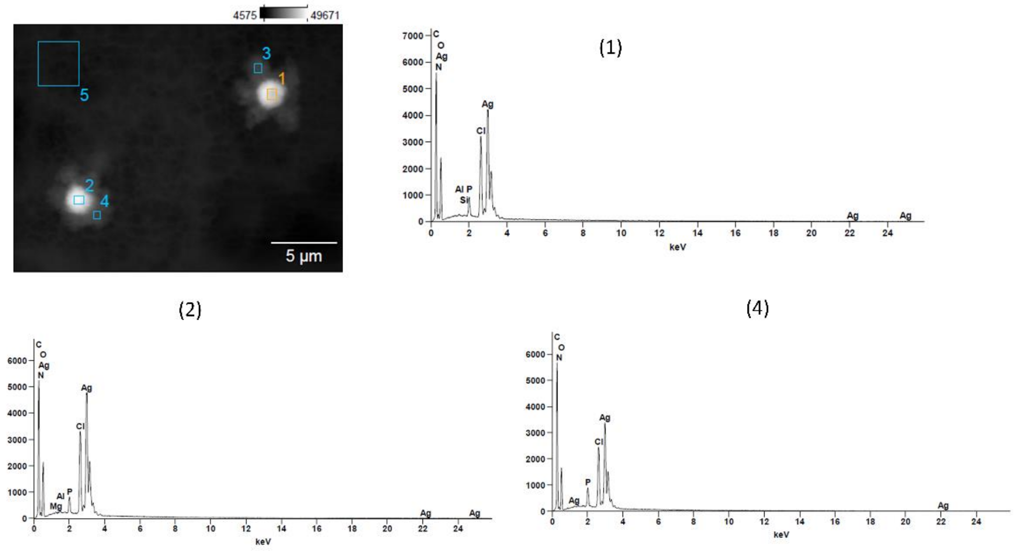

Figure S18. EDS-element mapping of **1**@AHSP.

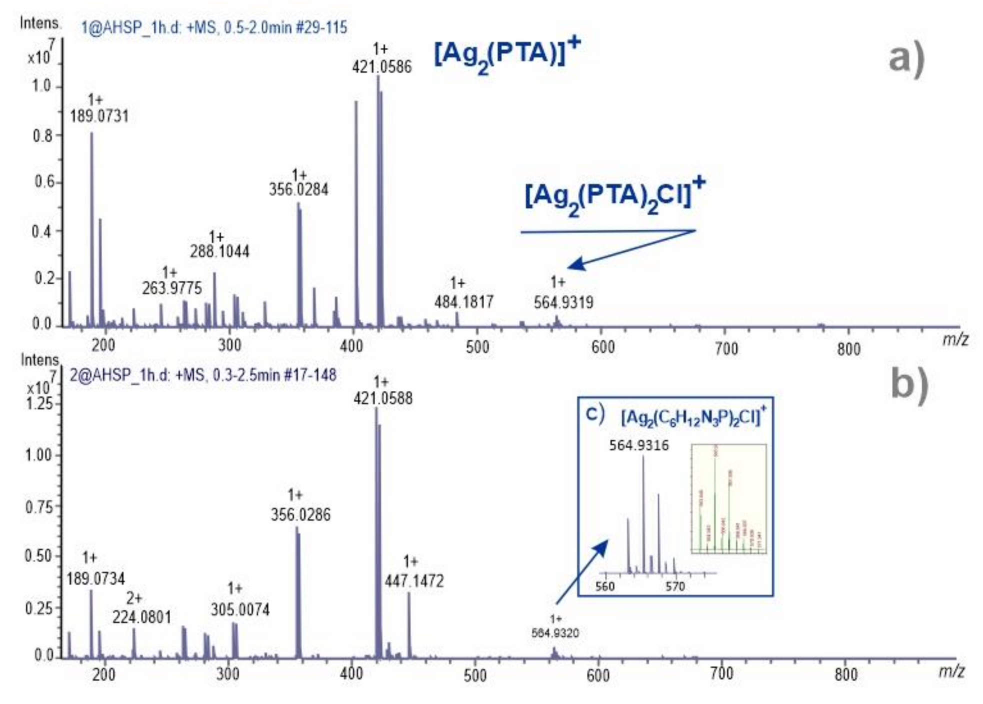

Figure S19. HR full scan mass spectra obtained in the ESI positive mode for solutions after immersing biocomposites for 1 h in water: a) **1**@AHSP; b) **2**@AHSP. The group of peaks centered at *m/z* 564.9316 display an isotopic distribution pattern that agrees well with that calculated for $[Ag_2(C_6H_{12}N_3P)_2Cl]^+$ as shown in c).

Despite washing procedures, a residual concentration of Cl^- ions is probably present in polymer matrix. It is known that Ag^+ ions in pure solvent tend to adopt tetrahedral geometry.[S1] However, this tendency changes in the presence of heteroleptic ligands due to *s-d* hybridization, and the formation of very stable $[AgL_2]^+$ species occurs.[S2] Based on these results, we can conclude that a trace amount of chloride anion plays an important role in the solution speciation of these compounds. This particular ion in the solution can be strictly related to the antimicrobial activity of these compounds and their composite materials. Probably, in a cell medium, the formation of $[Ag_2(PTA)_2Cl]^+$ species also occurs and plays a significant role in the mechanism of antimicrobial action. However, more detailed studies are required to confirm the role of this species in the antimicrobial action of compounds **1** and **2**. Coordination of chloride ligand can significantly increase lipophilicity, enhance permeation through the bacterial lipid layer, and cause death by bacteria.

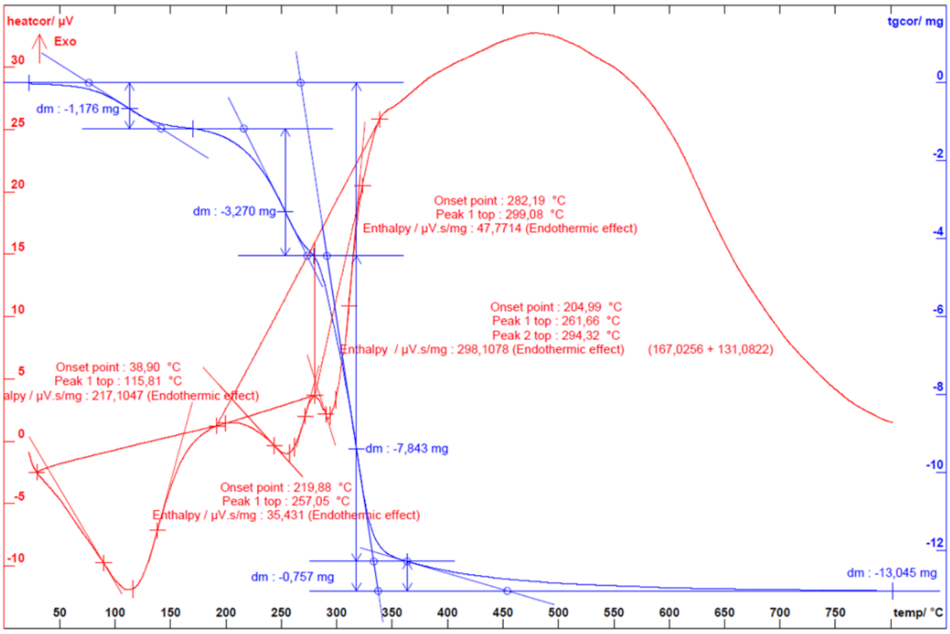

Figure S20. TGA data of the starting AHSP.

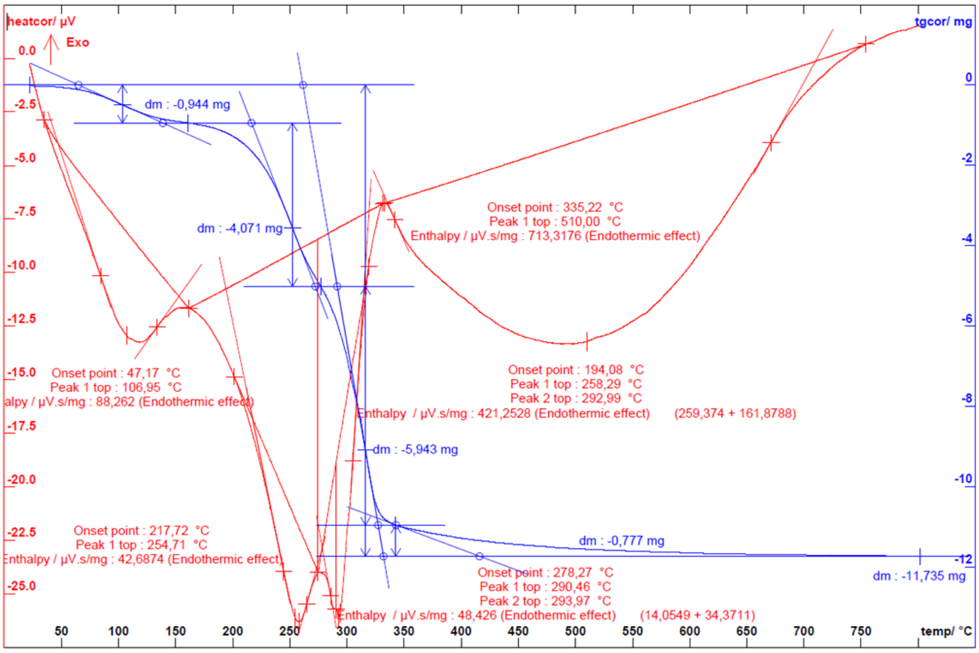

Figure S21. TGA data of **1**@AHSP.

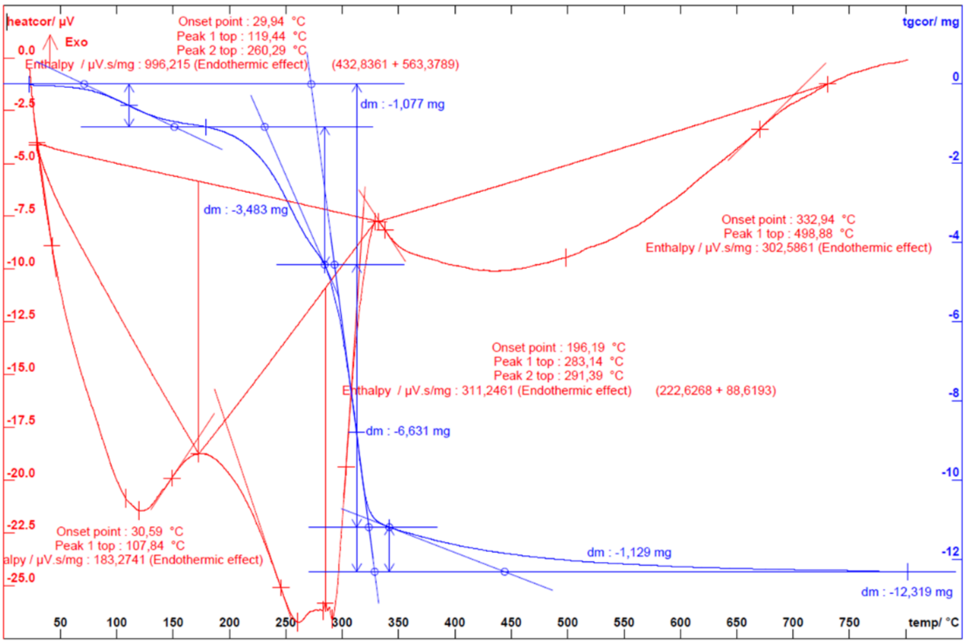

Figure S22. TGA plot of **2**@AHSP.

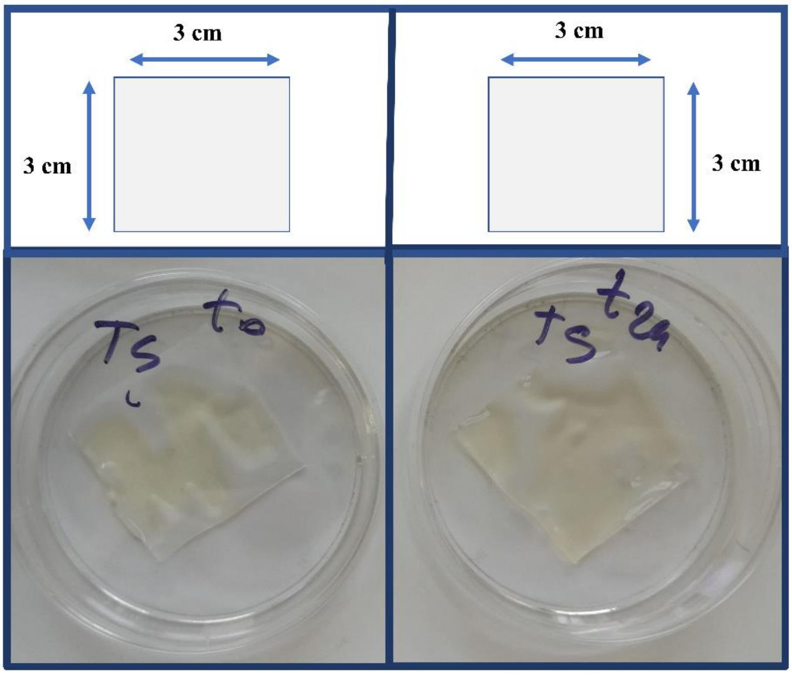

Figure S23. Examples of AHSP samples used for antiviral, antibacterial, and antifungal assays at different time intervals (t_0 and t_{24}).

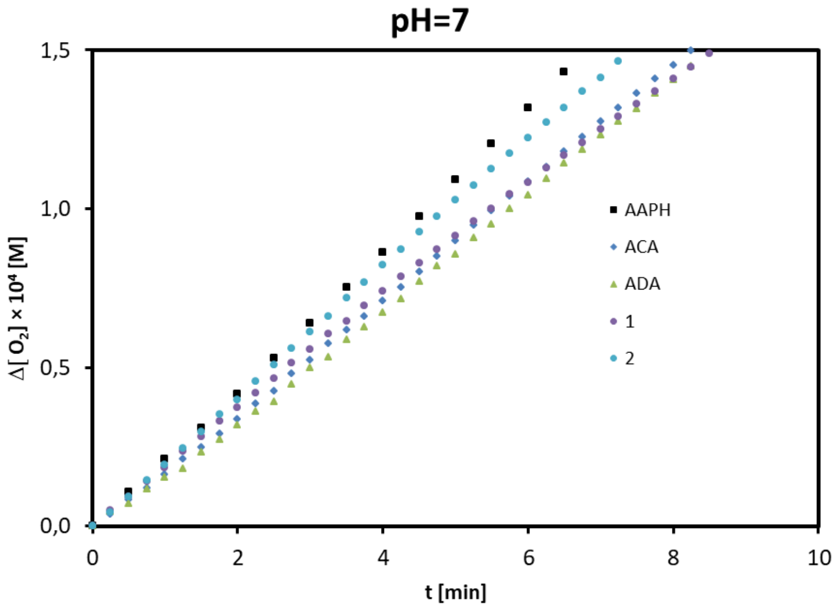

Figure S24. Oxygen uptake for autoxidation of micellar system initiated with AAPH at 37 °C, pH 7.0. Autoxidation in the absence (AAPH, blank) or presence of tested compounds (1 μM): Haca, H_2ada, **1**, **2**. Final concentration: LinMe 2.74 mM, Triton X-100 8 mM. Final results are determined based on four series of results obtained in two independent experiments (each experiment provided two series of results). Presented data are the most similar to the arithmetic average of 4 series of measurement data. The standard deviation was calculated and is 5-15%; however, the error bars are not included in the chart for clarity.

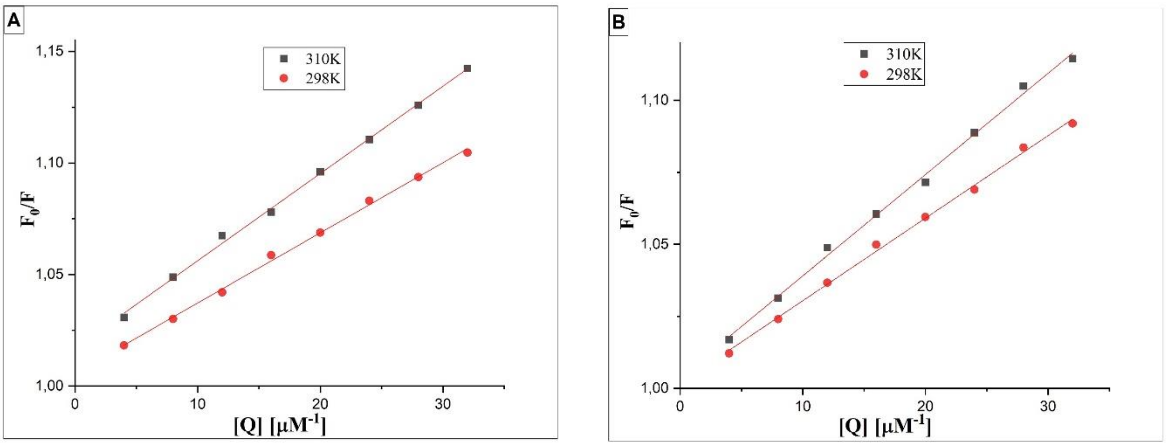

Figure S25. Stern-Volmer plots of the **1**-HSA (A) and **2**-HSA (B) systems at 310 and 298 K (λ_{ex} = 280 nm).

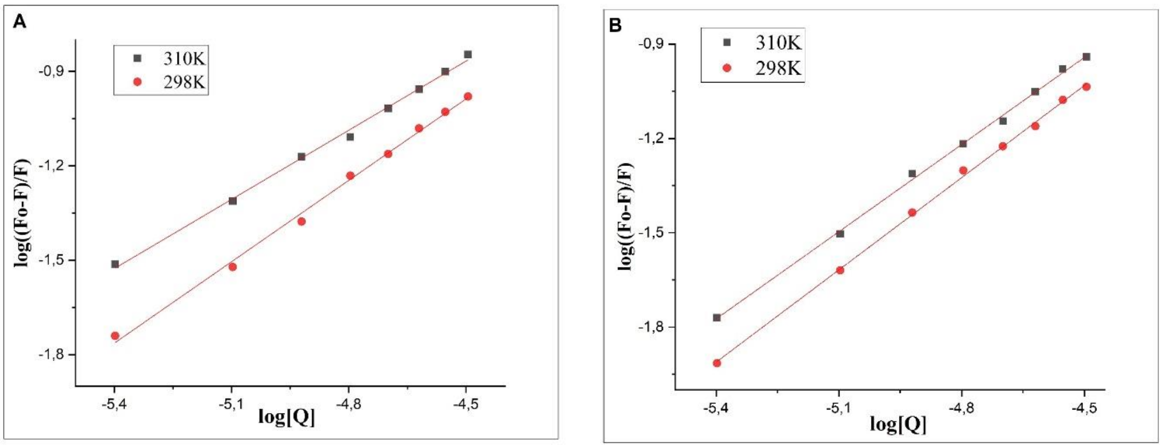

Figure S26. Double logarithm regression plots for quenching of HSA by **1** (A) and **2** (B) at 310 and 298 K (λ_{ex} = 280 nm).

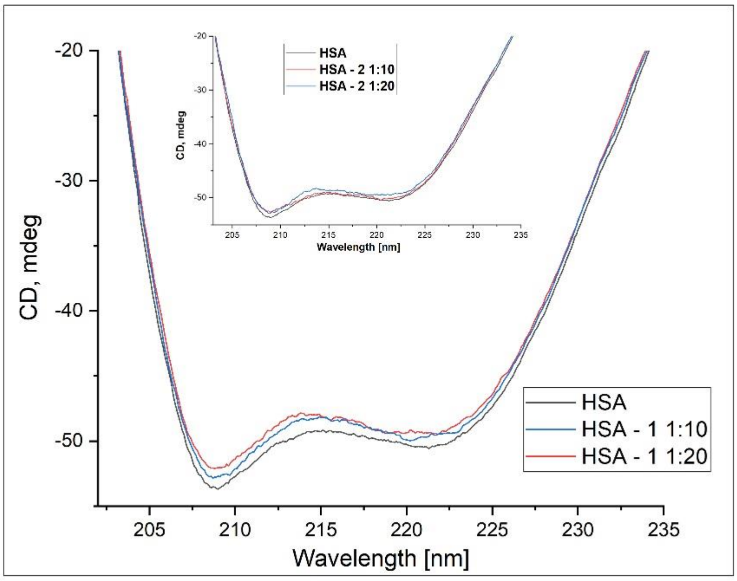

Figure S27. Effect of **1** and **2** (inset) concentration on CD spectra of HSA. Concentration of HSA, 4 μM, **1**-HSA or **2**-HSA was incubated for 24 h at 37 ° C at molar ratio 10:1 and 20:1.

Materials and Methods

M1. Materials, Reagents and General Methods. List of chemicals used: Ag_2O (Sigma-Aldrich), 1,3,5-triaza-7-phospaadamantane (Sigma-Aldrich), 3-adamantanecarboxylic acid (Sigma-Aldrich), 1,3-adamantanedicarboxylic acid (Sigma-Aldrich), methanol, corn starch (Sigma-Aldrich, S426), glycerine (Sigma-Aldrich), phosphate-buffered saline tablets (PBS, pH = 7.4, Sigma-Aldrich). Water, acetonitrile and formic acid LC-MS grade Optima® from Fisher Chemicals.

Elemental analyses were performed at the Faculty of Chemistry (the University of Wrocław) on the Elemental Analyser Vario ELCube (Elementar Analysen Systeme GmbH, Germany). For mass spectrometry analyses, Data were recorded at the Mass Spectrometry Facility at CQE. Node IST-RNEM (Portuguese Mass Spectrometry Network): high resolution mass spectra were obtained on a QqTOF Impact II™ mass spectrometer (Bruker Daltonics, Bremen Germany), equipped with an ESI source operating in the positive mode. Internal calibration of the mass analyzer was achieved with an ammonium formate solution introduced to the ion source via a 20 μL loop at the beginning of each analysis, using a six-port valve. Calibration was then performed using a high-precision calibration mode (HPC). The full scan mass spectra were acquired over a mass range of 100-1000 *m/z* at a spectra rate of 1 Hz. Data was processed using the Data Analysis 5.2 software. Low resolution mass spectra were acquired on a LCQ Fleet™ ion trap mass spectrometer (Thermo Fisher Scientific, Sao Jose, CA, USA), with an ESI ion source. The optimized parameters are as follow: spray voltage, ±4.5 kV; capillary voltage, 16/–18 V; tube lens offset, –63/58 V; sheath gas (N_2), 80 arbitrary units; auxiliary gas (N_2), 5 arbitrary units; capillary temperature, 270 ºC. MS^2 spectra were obtained by Collision Induced Dissociation (CID) experiments with an isolation window of 4-9 Da, a collision energy varying between 25 and 32%, and with an activation time of 30 ms. Spectra typically correspond to an average of 20–35 scans, and were recorded in the range between 100-2000 Da. Acquisition and data processing Data acquisition and processing were performed using the Xcalibur 2.2 SP1.48 and Mzmine[S3] software. The NMR spectra were measured at ambient temperature on a Bruker 500/600 AMX spectrometer. The NMR data were collected in D_2O and the ^1H chemical shifts (δ) are expressed in ppm relative to $Si(Me)_4$, while the $\delta(^{31}P)$ shifts are relative to an external H_3PO_4 (85% aqueous solution). The IR spectra were measured on a BIO-RAD FTS3000MX spectrophotometer in the range from 400 to 4000 cm^{-1} (KBr discs). The ICP-OES measurements were made on an iCAP 7400 ICP-OES emission spectrometer (model DUO, Thermo Scientific, manufactured in 2018), with sample excitation at the temperature of inductively coupled argon plasma (approx. 10,000K). The spectrometer is equipped with an ASX 280 autosampler (Teledyne), a classic pneumatic concentric nebulizer was used for nebulization. Solutions containing Ag^+ in the range of 0.01 - 1.0 mg/L were used for calibration.The emission was recorded for the 328.068 nm and 338.289 nm lines in the axial plasma observation mode, three times for each solution. The instrument limits of detection (LoD) are: LoD (328.068nm) = 0.0026mg/L and LoD (338.289nm) = 0.0022mg/L

M2. Thickness and Flatness Measurements of 1@AHSP and 2@AHSP. Biofilms thickness was measured by a digital caliper (Absolute Digimatic) using the square cut samples of **1**@AHSP and **2**@AHSP. The samples were divided into three different sections and each section was measured (L1) by caliper and cut. Each strap was then measured again (L2). The percent of constriction of both materials was calculated according to the following equation: %Constriction = [(L1−L2)/L2]×100%.

M3. Cell Lines and Media. A549 - human lung carcinoma (ATCC, No CCL-185 ™) and HeLa -human cervix carcinoma (ATCC, No CCL-2 ™) cell lines from American Type Culture Collection—ATCC (Rockville, MD, USA) were used for determination of virucidal properties. Dulbecco's Modified Eagle's Medium - DMEM (Lonza, Basel, Switzerland) served as substrate and was supplemented with fetal bovine serum (FBS, 10%) and L-glutamine (4 mM) (Biological Industries, Kibbutz Beit-Haemek, Israel), 100 U/mL of penicillin, and 100 μg/L of streptomycin (Sigma-Aldrich, Munich, Germany).

M4. Antiviral Assays. The solutions of **1** and **2** in 0.3 mg/mL were tested using herpes simplex virus type 1 (HSV-1 - ATCC® VR-1493™) and human adenovirus 5 (HAdv-5, virus-strain Adenoid 75, ATCC VR-5™). The assays were performed according to the standard EN 14476 [Chemical Disinfectants and Antiseptics − Quantitative Suspension Test for the Evaluation of Virucidal Activity in the Medical Area − Test Method and Requirements (Phase2/Step 1); European Committee for Standardization: Brussels, Belgium, 2013]. This standard describes a quantitative suspension test for the evaluation of virucidal activity in the medical area (Phase 2/Step 1). One part by volume of test virus suspension (0.1 mL of 1×10^{12} $TCID_{50}$ HAd-36 virus or HSV-1), one part by volume of interfering substance (0.1 mL of PBS), and eight parts by volume of disinfectant (investigated substances in a concentration 0.3 mg/mL) were mixed. At specified contact times (60 min), aliquots were taken, and serial dilutions from 10^{-1} to 10^{-12} of each mixture were prepared. Then, 50 μL of each dilution was added to the microtiter plate containing a monolayer of confluent A549 or HeLa cells. Each dilution was tested in eight repeats. The plates were observed daily for up to 4 days for the development of viral cytopathic effect (CPE), using an inverted microscope (Olympus Corp., Hamburg, Germany; Axio Observer, Carl Zeiss MicroImaging GmbH). Then, a residual infectivity was determined. According to the standard, a disinfectant is considered as having virucidal effectiveness if

within the recommended exposure time the titer is reduced by ≥4 log10 steps, corresponding to ≥ 99.99% inactivation).

Calculation of the infective dose $TCID_{50}$/mL was calculated by the Spearman and Karber method using the following formula:

$$\log_{10}TCID_{50} = x_0 - 0.5 + \Sigma\, r/n$$

wherein x_0 = log 10 of the lowest dilution with 100% positive reaction, r = number of positive determinations of lowest dilution step with 100% positive and all higher positive dilution steps, and n = number of determinations for each dilution step, $TCID_{50}$ = tissue culture infectious dose.

The polymer biofilms doped with **1** and **2** were also tested using the same viruses and medium as above, according to the standard ISO 21702 [Measurement of the antiviral activity on plastics and other non-porous surfaces; ISO, Switzerland, Geneva, 2019]. This document specifies proper protocols for measuring antiviral activity on plastics and other non-porous surfaces of antiviral-treated products against specified viruses. For this purpose, a sample of the test material treated with SCDLP (soybean casein digest broth with lecithin and polyoxyethylene sorbitan monooleate) neutralizer was placed in a sterile Petri dish. On the sample prepared in this way, 400 µL of the test virus was applied and covered with a 2×3 cm layer of thermoplastic. The sample was incubated at 25 °C for 24 h at 90% humidity. After this time, the virus was collected by pipette. The study was conducted in 3 replicates. In parallel, as a control, the whole procedure was repeated using a control disc as a non-virucidal material of the same dimensions as the test sample, also in triplicate. In addition, immediately after virus inoculation onto the control disc, virus titers were tested (3 replicates).

Given that ISO 21702 permits the use of the $TCID_{50}$ method described in EN 14476 instead of the plaque test, serial dilutions up to 10^{-12} of the viruses collected from each disc were prepared and inoculated into 96-wells polystyrene plate. They were observed for the development of viral cytopathic effect (CPE) that is an indicator of virus survival.

M5. Antimicrobial Activity. Determination of MICs for **1** and **2** against bacteria and yeasts was performed as described before.[S4] Briefly, the method of serial dilutions in the antibiotic broth (AB) described by Grove and Randall[S5] was used. The tests were performed on two reference strains, i.e. *Staphylococcus aureus* PCM 2054 (= ATCC 25923) and *Escherichia coli* PCM 2057 (= ATCC 25922), as well as four field bacterial (*Pseudomonas aeruginosa*, *Bacillus cereus*, *Bordetella bronchiseptica*, *Klebsiella pneumoniae*) and two fungal (*Candida albicans*, *Cryptococcus neoformans*) isolates. Overnight cultures of the microorganisms in AB were

subsequently diluted 1:100 (as for *C. neoformans*) or 1:1000 (in the case of the remaining strains)). Final concentrations of **1** and **2** in AB were as follows: 60, 50, 40, 30, 20, 10, 9, 8, 7, 6, 5, 4, 3, 2, and 1 μg mL^{-1}. Broth sterility and growth controls were also performed. Inoculated plates were then incubated at 37 °C for 24 h. This procedure was carried out three times and with two technical replicates each. The minimum inhibitory concentration (MIC, μg mL^{-1}) was defined as the lowest concentration of the compound that fully inhibited the growth of microorganisms. For comparison, the MIC values were normalized for the molar content of silver and are presented in a nmol mL^{-1} scale. Additionally, activities of $AgNO_3$ and the ligands (PTA, Haca, and H_2adc) were also tested.

M6. Surface Activity of Starch Polymers Doped by 1 and 2. This investigation of surface activity against bacteria and yeasts was based on an ASTM E2180 protocol "Standard Testing Method for Determining Antimicrobial Activity in Polymeric or Hydrophobic Materials" with few modifications. Overnight cultures of *E. coli, S. aureus, P. aeruginosa,* and *C. albicans* were used to prepare inocula (each microbial suspension contained approximately 1×10^8 cells per 1mL of saline). The obtained suspensions were diluted 1:100 in tryptic soy broth (TSB), and 0.5 mL aliquots of the dilution were placed onto the surface of square-shaped starch polymers (3×3 cm, 1 mm thick). Four squares were used for each strain and compound: two blank ones (T0 and T24, corresponding to 0 and 24 h, respectively), and two (duplicate) compound-loaded squares, intended for a 24 h incubation (T24). Immediately after inoculation, the squares assigned to the T0 analysis were transferred into Falcon tubes containing 40 mL of D/E Neutralizing Broth and vortexed for 3 min. The obtained suspensions of microorganisms were then serially diluted in saline, and inoculated onto plates containing tryptic soy agar (TSA). The plates were incubated overnight, and the number of colony forming units (CFUs) on each plate was estimated. Based on the number of CFUs, the number of living bacterial cells detached from each polymer square was calculated. The procedure was carried out in duplicate, and the results were expressed as an arithmetic mean of colony forming units (CFUs) grown on a given medium, which, in turn, were converted into the logarithm or percentage of the microbial reduction. The T24 squares were incubated for 24 h at 37°C in a humid chamber. After the incubation period, the squares were proceeded as described for T0.

M7. Autoxidation Measurements. The antioxidant behavior of **1**, **2**, and free ligands (Haca and H_2ada) was measured by monitoring the rate of peroxidation in a heterogeneous model system (micelles, Triton X-100). The uptake of dissolved oxygen in micelles during peroxidation was carried out at 37 °C by using a Biological Oxygen Monitor equipped with a

Clark-type oxygen electrode. To a glass chamber containing a magnetic stirrer, 5 mL of micelles (pH 7.0) was added and then saturated with oxygen. Next, the electrodes were placed inside the chambers and a peroxidation was initiated by injection of the AAPH solution (final concentration: 10 mM). After 10% of oxygen consumption, 10 μL of the solution of studied compounds was added (final concentration was 1 μM). The influence of tested compounds on the peroxidation was determined graphically. Due to the neutrality of **1** to peroxidation of micelles or weak retardation activity of **1**, Haca, and H_2ada, the kinetic parameters were not determined.

M8. Silver Release and Stability Studies. Silver concentration and ion release process from the **1**@AHSP and **2**@AHSP materials were monitored for 24 h in a medium representing simulated physiological conditions (phosphate buffered saline (PBS), pH 7.4, 10 mL). The concentration of silver release from **1**@AHSP and **2**@AHSP was established by ICP-OES. Stock solution samples were stored during 24 h at room temperature. Small aliquots (20 μL) of samples were withdrawn every 4 h and replaced with the same volume of PBS. After the analysis, the polymer samples were dried, weighted, and analyzed by IR spectroscopy. M9.

M9. Interaction of 1 and 2 with Human Serum Albumin. Human serum albumin was purchased from Sigma-Aldrich Co. (Catalog No. A3782, ≥99% purity). The protein stock solution with a concentration 2 μM was prepared in PBS buffer (pH=7.40). Metal complexes stock solutions with a concentration 833 μM were prepared by dissolution in double distilled water. Human serum albumin concentration was determined spectrophotometrically using $\varepsilon_{280} = 36600 M^{-1} cm^{-1}$[S6]. Fluorescence measurements were performed on Jasco 8200 spectrofluorometer using a 1.0 cm quartz cell. The excitation/emission slits were set at 5.0 nm. Spectra were recorded in the range of 300 - 500 nm at the excitation wavelength of 280 nm. PBS buffer was taken as a blank. The fluorescence studies were done by titration of 2 ml HSA with 10 μl appropriate silver complex. The samples were measured after 5 mins incubation at 298K and 310K. The final spectra are an average of 3 scans. Circular dichroism measurements were performed on Jasco J-1500 CD spectrometer using 0.1 cm quartz cell. A bandwidth of 1 nm, a response time of 2 s and scanning speed at 50 nm/min were fixed to obtain final spectra as an average of 3 scans. The final concentration of the protein was 2 μM and molar ratio [HSA]:[complex] = 1:0, 1:10 and 1:20. Spectra were recorded after 24h incubation at 310K in the range of 200-250nm.

Interaction of 1 and 2 with Human Serum Albumin. Fluorescence spectroscopy is one of the most effective methods to investigate the interaction of biomolecules with small particles

and determine the structural changes and binding parameters between them. We used this technique to determine the quenching mechanism of HSA fluorescence by **1** and **2** and calculate the binding and thermodynamic parameters of the systems. Human serum albumin shows a strong fluorescence signal at 335.5 nm after excitation at 280 nm. Under tested conditions, the analysed compounds do not show any fluorescence signal. Titration with **1** and **2** causes a slight quenching of the protein fluorescence and blueshift of the emission peak (2 nm). This behavior indicates the interaction between human serum albumin and metal compounds and some conformational changes of the protein.

To determine the quenching parameters of the systems, number of binding sites and association constant between HSA and the ligands, the equations 1–3 were used. All data are collected in Table S1.

$$\frac{F_0}{F} = K_{SV}[Q] + 1 \qquad \text{Eq. 1}^{S7}$$

$$K_q = {}^{K_{SV}}/_{\tau} \qquad \text{Eq. 2}$$

$$log\frac{(F_0 - F)}{F} = logK_a + nlog[Q] \qquad \text{Eq. 3}^{S8}$$

Herein, F_0 and F are the relative fluorescence intensities of protein in the absence and presence of the quencher, respectively, K_{sv} is the Stern–Volmer quenching constant, K_q is the quenching rate constant, τ refers to an average lifetime of the protein (10 ns), K_a is the binding constant, n is the number of binding sites, and [Q] stands for the quencher concentration.

The calculated Stern-Volmer quenching constants (K_{sv}) are directly correlated with temperature. The values are 3.09×10^3 and 4.04×10^3 M^{-1} for **1** and 2.91×10^3 and 3.54×10^3 M^{-1} for **2** at 298 and 310 K, respectively. These point at dynamic quenching mechanisms of the protein which involve fluorophore and a quencher collisions during transient existence of the excited state. But taking into account the maximum scatter collision quenching constant of various quenchers with the biopolymer ($K_q = 2.0\times10^{10}$ M^{-1} s^{-1}) this indicates the contribution of static quenching mechanism of the protein fluorescence by the compounds and refers to the formation of HSA-**1** or HSA-**2** complexes (appropriate **1** and **2** values of 3.09, 4.04 and 2.91, 3.54 are in the order of 10^{11} M^{-1} s^{-1}).[S9]

From the data collected in Table S1 and Figures S25–S26 it is clear that the binding constants (K_a) decrease with temperature raising when the protein interacts with both compounds. Such relationships indicate that **1** and **2** form unstable complexes that undergo partial decomposition at higher temperatures. The n values approximate close to 1 in both systems indicate that only

one binding site in protein is accessible for the complex. The binding constants of the systems are in the order of 10^2–10^3 M^{-1} and are comparable with the value of HSA – cisplatin (K_a = 8.52×10^2 M^{-1}).[S10] For comparison, the association constants of the ruthenium complex tested in clinical trials KP1339 is K_a = 4.57×10^4 M^{-1}.[S11]

Using the Equations 4–6 and the Ross & Subramanian theory the thermodynamic parameters and acting forces between **1** or **2** and HSA were determined:

$$ln\frac{Ka_2}{Ka_1} = \frac{\Delta H^0}{R}\left(\frac{1}{T_1} - \frac{1}{T_2}\right) \quad \text{Eq. 4}$$

$$\Delta G^0 = -RTlnK_a \quad \text{Eq. 5}$$

$$\Delta S^0 = \frac{\Delta H^0 - \Delta G^0}{T} \quad \text{Eq. 6}$$

In the equations, ΔH^0, ΔS^0, ΔG^0 are the enthalpy, free energy and entropy, respectively, while K_a is the binding constant at the corresponding temperature, T stands for the experimental temperature [K], and R is the gas constant.

In general, four types of bonding forces between small molecules and biological macromolecules may be present in the interaction. These include electrostatic (Coulomb) forces, hydrophobic and ionic interactions, as well as Van der Waals forces and hydrogen bonds. Furthermore, contribution of metal ions in the interaction is also possible in the case of coordination compounds. The obtained data show that Van der Waals interactions and hydrogen bonds as the main forces in the HSA-**1** and HSA-**2** systems because of the both negative ΔH^0 and ΔS^0. Additionally, negative ΔG^0 values prove spontaneous interaction between molecules.

Table S1. Binding and thermodynamic parameters for the interaction of HSA with the studied compounds at different temperatures

	T [K]	K_{sv} [M^{-1}] ×10^3 (±SD)	K_q [$M^{-1}s^{-1}$] ×10^{11}(±SD)	K_a [M^{-1}] ×10^2(±SD)	n(±SD)	ΔH [kJ/mol] (±SD)	ΔS [J/mol·K] (±SD)	ΔG [kJ/mol] (±SD)
**1**	298	3.09(±0.03)	3.09(±0.03)	7.78(±0.02)	0.86(±0.01)	−79.98(±0.18)	−213.05(±0.25)	−16.49(±0.05)
	310	4.04(±0.02)	4.04(±0.02)	2.23(±0.04)	0.72(±0.02)			−13.94(±0.06)
**2**	298	2.91(±0.06)	2.91(±0.06)	23.3(±0.05)	0.98(±0.03)	−22.48(±0.21)	−10.97(±0.24)	−19.21(±0.04)
	310	3.54(±0.04)	3.54(±0.04)	16.4(±0.06)	0.92(±0.02)			−19.08(±0.06)

Circular dichroism (CD) spectroscopy is a widely used technique for studying protein conformations. We used the CD technique to determine the effect of **1** and **2** on the HSA secondary structure. The CD spectrum in the far UV CD region (190−250 nm) is characterised by negative bands at 209 and 221 nm, assigned to its α-helical structure and π→π* and n→π* transitions.[S12] Under the experimental conditions, **1** and **2** do not generate a CD signal. Human serum albumin is mostly an α-helical protein. The α-helical content of free HSA at 209 nm was found to be 66.5%. A decrease in α-helicity to 64.4/64.3% without changes in bands position was observed after addition of **1** or **2** at the molar ratio of [HSA]/[Ag] = 1:20. These changes arise from the interaction between serum albumin and silver compounds. The obtained results were analysed by the CDPro software and are summarized in Table S2. CD spectra are shown in Figure S27, SI.

Table S2. The percentage of HSA secondary structure in the absence and presence of **1** and **2** calculated with the CDPro software.

	α – helix (%)	β – sheet (%)	β – turn (%)	Random coil (%)
HSA	66.5 (±0.10)	2.7 (±0.10)	9.9 (±0.10)	20.7 (±0.10)
HSA–**1** 1:10	65.7 (±0.12)	3.0 (±0.12)	10.6 (±0.12)	20.4 (±0.12)
HSA–**1** 1:20	64.4 (±0.14)	2.2 (±0.14)	11.6 (±0.14)	21.2 (±0.14)
HSA–**2** 1:10	65.8 (±0.10)	2.8 (±0.10)	10.1 (±0.10)	20.8 (±0.10)
HSA–**2** 1:20	64.3 (±0.12)	2.1 (±0.12)	11.6 (±0.12)	21.3 (±0.12)

M10. Computational Studies. Computational docking of diverse moieties originating from the speciation of the investigated coordination polymers was carried out according to the following procedure. Structures of ligands (1,3,5-triaza-7-phosphaadamantane, 1-adamantanecarboxylic acid) and fragments of the coordination network were generated from the experimental X-ray data of **1** using a CCDC Mercury 2020.1 program. The compounds of the AgL_2 type were linearized to represent the s-d hybridization of the silver atom. The structures of two macromolecules chosen for this study, namely the M2 proton channel of influenza A virus and the p7 cationic channel of hepatitis C virus, were taken from the Protein Data Bank[S13] (deposits 6BKK and 2M6X, respectively). The protein and tentative ligand structures were processed using AutoDock Tools component of the MGLTools 1.5.7 suite[S14] – polar hydrogen atoms were added, Gasteiger atomic charges and AutoDock4 atom types were assigned. Detection of rotatable bonds was carried out for the ligands. The docking was

performed with the AutoDock Vina 1.1.2 program[S15] requesting up to 12 binding modes to be generated. The search exhaustiveness parameter was set to 18. The search boxes were centered on the experimental coordinates of the amantadine ligand in case of 6BKK, and on the barycenter of the protein in case of 2M6X. The respective cubic box sizes were 30 and 60 Å. The results were analyzed with the help of the AutoDock Tools program. Additional docking runs used amantadine as the ligand, so that the docking accuracy could be assessed against the experimental location of the adamantane-like binding site in 6BKK.

M11. Moisture Uptake. First, the squares of AHSP, **1**@AHSP and **2**@AHSP were weighed and put into a desiccator with a drying agent for 24 h at ambient temperature. After 24 h, each square of biofilm was weighed again. The moisture content was calculated using the following equation: %moisture content = [(W1 - W2)/W2]×100% (W1-initial weight, W2-final weight). Then the squares were transferred to a desiccator filled with a 100 mL saturated solution of KCl. The potassium chloride was used to provide ca. 84% humidity. The biofilms were kept under this condition in a desiccator until the constant weight was observed. Moisture uptake was calculated as follows: %moisture uptake = [(W2 - W1)/W1]×100%.[S16]

Table S3. Selected bond lengths (Å) for **1**.

Ag1–P1	2.339(2)	N2–C12	1.457(11)
Ag1–O1	2.448(6)	N2–C16	1.483(10)
Ag1–N2[a]	2.458(7)	N2–C17	1.485(10)
Ag1–O2	2.449(6)	N3–C14	1.481(10)
P1–C12	1.834(8)	N3–C15	1.465(9)
P1–C13	1.837(8)	N3–C16	1.467(10)
P1–C14	1.837(8)	C3–C9	1.537(11)
C1–O1	1.261(10)	C4–C10	1.548(11)
C1–C2	1.526(13)	C5–C11	1.509(12)
C1–O2	1.270(10)	C6–C9	1.523(11)
N1–C13	1.481(10)	C6–C10	1.516(11)
N1–C15	1.460(9)	C7–C10	1.541(12)
N1–C17	1.473(10)	C7–C11	1.537(14)
C2–C3	1.539(11)	C8–C9	1.535(12)
C2–C4	1.523(11)	C8–C11	1.500(14)
C2–C5	1.558(13)		

[a] +x, 3/2 – y, –1/2 + z

Table S4. Selected bond angles (°) for **1**.

P1–Ag1–O1	142.26(15)	C16–N2–Ag1[b]	108.8(5)
P1–Ag1–N2[a]	117.20(17)	C16–N2–C17	107.2(6)
P1–Ag1–O2	139.11(16)	C17–N2–Ag1[b]	109.6(5)
O1–Ag1–N2[a]	92.5(2)	C1–O2–Ag1	92.3(5)
O1–Ag1–O2	53.5(2)	C15–N3–C14	111.6(6)
O2–Ag1–N2[a]	92.2(2)	C15–N3–C16	108.7(6)
C12–P1–Ag1	121.6(3)	C16–N3–C14	111.4(6)
C12–P1–C13	97.1(4)	C9–C3–C2	109.6(7)
C12–P1–C14	96.8(4)	C2–C4–C10	109.8(7)
C13–P1–Ag1	119.4(3)	C11–C5–C2	109.7(8)
C14–P1–Ag1	118.7(3)	C10–C6–C9	110.2(7)
C14–P1–C13	98.1(4)	C11–C7–C10	108.6(7)
O1–C1–C2	119.9(7)	C11–C8–C9	109.0(7)
O1–C1–O2	121.2(8)	C6–C9–C3	109.7(7)
O2–C1–C2	118.8(7)	C6–C9–C8	109.7(7)
C15–N1–C13	111.6(6)	C8–C9–C3	109.0(7)
C15–N1–C17	108.6(6)	C6–C10–C4	110.0(7)
C17–N1–C13	111.3(6)	C6–C10–C7	109.3(7)
C1–O1–Ag1	92.6(5)	C7–C10–C4	108.4(7)
C1–C2–C3	110.9(7)	C5–C11–C7	108.3(8)
C1–C2–C5	107.5(7)	C8–C11–C5	111.4(8)
C3–C2–C5	108.7(7)	C8–C11–C7	111.0(8)
C4–C2–C1	111.7(6)	N2–C12–P1	115.6(6)
C4–C2–C3	110.0(8)	N1–C13–P1	112.4(5)
C4–C2–C5	107.9(7)	N3–C14–P1	112.3(5)
C12–N2–Ag1[b]	111.3(5)	N1–C15–N3	113.7(7)
C12–N2–C16	109.8(6)	N3–C16–N2	114.4(6)
C12–N2–C17	110.1(6)	N1–C17–N2	114.4(6)

[a] x, 3/2 – y, –1/2 + z; [b] x,3/2 – y,1/2 + z

Table S5. Hydrogen bonding parameters for **1**.

O–H···X	*d*(O–H), Å	*d*(H···X), Å	*d*(O···X), Å	∠(O–H···X), °

O1W–H11···O2	0.960(5)	1.78(4)	2.713(9)	163(11)
O1W–H12···O1[a]	0.960(5)	1.81(4)	2.728(9)	158(9)
O2W–H22···O4W	0.961(5)	1.76(4)	2.71(2)	167(11)
O2W–H21···O1W	0.960(5)	1.74(4)	2.668(14)	160(11)
O3W–H31···O2W	0.960(5)	1.75(6)	2.645(18)	153(10)
O3W–H32···O1W[b]	0.960(5)	1.86(5)	2.771(18)	158(11)
O4W–H41···O1[c]	0.960(5)	2.02(3)	2.965(14)	169(11)
O4W–H42···O2[b]	0.960(5)	2.07(3)	2.998(14)	163(9)
O5W–H51···N3	0.960(5)	1.90(3)	2.835(9)	165(10)
O5W–H52···N1[d]	0.960(5)	1.91(3)	2.844(9)	164(10)

[a] x, –1 + y, z; [b] x, 1/2 – y, –1/2 + z; [c] x, 3/2 – y, –1/2 + z; [d] x, 1 + y, z

Table S6. Selected bond lengths (Å) for **2**.

N1A–Ag2[a]	2.389(3)	P2B–C16	1.535(7)
N1A–C11	1.548(6)	N3B–Ag2	2.379(3)
N1A–C12	1.544(6)	N3B–C16	1.722(6)
N1A–C13	1.557(5)	N3B–C16[c]	1.722(6)
P1A–Ag1	2.364(2)	N3B–C17	1.666(14)
P1A–C10	1.727(8)	C1–O1[b]	1.264(5)
P1A–C11[b]	1.713(6)	C1–O1	1.264(5)
P1A–C11	1.713(6)	C1–C2	1.542(9)
P1B–Ag2[a]	2.389(3)	C2–C3[b]	1.529(6)
P1B–C11	1.548(6)	C2–C3	1.529(6)
P1B–C12	1.544(6)	C2–C4	1.539(8)
P1B–C13	1.557(5)	N2–C10	1.473(10)
N1B–Ag1	2.364(2)	N2–C12	1.463(7)
N1B–C10	1.727(8)	N2–C12[b]	1.463(7)
N1B–C11[b]	1.713(6)	O2–C9	1.186(11)
N1B–C11	1.713(6)	O2W–O3W	2.751(8)
N3A–Ag1	2.379(3)	C3–C5	1.532(7)
N3A–C14	1.574(6)	N4–C14	1.464(7)
N3A–C15	1.574(5)	N4–C14[c]	1.464(7)
N3A–C16	1.535(7)	N4–C17	1.458(15)
P2A–Ag2	2.379(3)	C4–C6	1.543(9)
P2A–C16	1.722(6)	C5–C7	1.523(6)
P2A–C16[c]	1.722(6)	C5–C8	1.532(7)
P2A–C17	1.666(14)	C6–C8	1.521(7)
P2B–Ag1	2.379(3)	C6–C8[b]	1.521(7)
P2B–C14	1.574(6)	C6–C9	1.534(10)
P2B–C15	1.574(5)		

[a] 1/2 + x, y,1/2 – z; [b] x, 1/2 – y, z; [c] x, –1/2 – y, z

Table S7. Selected bond angles (°) for **2**.

C11–N1A–Ag2[a]	110.5(3)	N1B–Ag1–P2B	119.77(8)
C11–N1A–C13	105.9(4)	N3A–Ag1–N3A[b]	104.12(15)
C12–N1A–Ag2[a]	116.6(3)	O12–C1–O1	122.6(6)
C12–N1A–C11	106.2(3)	O12–C1–C2	118.7(3)
C12–N1A–C13	105.3(4)	O1–C1–C2	118.7(3)
C13–N1A–Ag2[a]	111.6(3)	N1A4–Ag2–N1A[e]	105.22(15)
C10–P1A–Ag1	118.0(3)	P2A–Ag2–N1A[e]	117.88(8)
C11–P1A–Ag1	117.0(2)	P2A–Ag2–N1A[d]	117.88(8)
C112–P1A–Ag1	117.0(2)	C3–C2–C1	111.6(3)

C112–P1A–C10	100.1(2)	C32–C2–C1	111.6(3)
C11–P1A–C10	100.1(2)	C32–C2–C3	108.5(5)
C112–P1A–C11	101.6(4)	C3–C2–C4	109.0(4)
C11–P1B–Ag2[a]	110.5(3)	C32–C2–C4	109.0(4)
C11–P1B–C13	105.9(4)	C4–C2–C1	107.0(5)
C12–P1B–Ag2[a]	116.6(3)	C122–N2–C10	110.8(4)
C12–P1B–C11	106.2(3)	C12–N2–C10	110.8(4)
C12–P1B–C13	105.3(4)	C122–N2–C12	110.6(6)
C13–P1B–Ag2[a]	111.6(3)	C2–C3–C5	110.2(4)
C10–N1B–Ag1	118.0(3)	C143–N4–C14	111.1(7)
C11–N1B–Ag1	117.0(2)	C17–N4–C14	110.9(5)
C112–N1B–Ag1	117.0(2)	C17–N4–C14[c]	110.9(5)
C11–N1B–C10	100.1(2)	C2–C4–C6	109.9(5)
C112–N1B–C10	100.1(2)	C3–C5–C8	108.9(4)
C112–N1B–C11	101.6(4)	C7–C5–C3	109.5(5)
C14–N3A–Ag1	111.5(3)	C7–C5–C8	110.0(5)
C14–N3A–C15	103.1(4)	C4–C6–C9	106.6(6)
C15–N3A–Ag1	111.9(3)	C82–C6–C4	108.8(4)
C16–N3A–Ag1	117.0(3)	C8–C6–C4	108.8(4)
C16–N3A–C14	106.6(4)	C82–C6–C8	110.1(6)
C16–N3A–C15	105.7(4)	C8–C6–C9	111.2(4)
C16–P2A–Ag2	114.2(2)	C82–C6–C9	111.2(4)
C163–P2A–Ag2	114.2(2)	C52–C7–C5	109.1(5)
C16–P2A–C16[c]	99.7(4)	C6–C8–C5	109.9(4)
C17–P2A–Ag2	122.2(4)	O2–C9–O2[b]	118.9(11)
C17–P2A–C16	101.8(3)	O22–C9–C6	120.3(6)
C17–P2A–C16[c]	101.7(3)	O2–C9–C6	120.3(6)
C14–P2B–Ag1	111.5(3)	N2–C10–P1A	113.8(5)
C14–P2B–C15	103.1(4)	N2–C10–N1B	113.8(5)
C15–P2B–Ag1	111.9(3)	N1A–C11–P1A	117.1(4)
C16–P2B–Ag1	117.0(3)	P1B–C11–N1B	117.1(4)
C16–P2B–C14	106.6(4)	N2–C12–N1A	115.5(5)
C16–P2B–C15	105.7(4)	N2–C12–P1B	115.5(5)
C16–N3B–Ag2	114.2(2)	N1A–C13–N1A[b]	118.0(5)
C163–N3B–Ag2	114.2(2)	N4–C14–N3A	115.1(5)
C16–N3B–C16[c]	99.7(4)	N4–C14–P2B	115.1(5)
C17–N3B–Ag2	122.2(4)	N3A–C15–N3A[c]	119.1(5)
C17–N3B–C16	101.8(3)	N3A–C16–P2A	117.1(4)
C17–N3B–C16[c]	101.7(3)	P2B–C16–N3B	117.1(4)
P1A–Ag1–N3A[b]	119.77(8)	N4–C17–P2A	115.1(6)
P1A–Ag1–N3A	119.77(8)	N4–C17–N3B	115.1(6)

[a] 1/2 + x, y, 1/2 – z; [b] x, 1/2 – y, z; [c] x, –1/2 – y, z; [d] –1/2 + x, –1/2 – y, 1/2 – z; [e] –1/2 + x, y, 1/2 – z

Table S8. Hydrogen bonding parameters for **2**.

O–H···X	*d*(O–H), Å	*d*(H···X), Å	*d*(O···X), Å	∠(O–H···X), °
O1W–H11···O1	0.959(10)	1.926(17)	2.870(5)	168(5)
O2W–H21···O1	0.958(10)	1.834(14)	2.786(5)	172(5)
O3W–H31···O2	0.964(10)	2.55(5)	3.386(15)	146(6)
O4W–H41···O2	0.959(10)	1.977(17)	2.933(13)	174.1(19)

Table S9. Physicochemical parameters of tested biomaterials.

Biopolymer	Thickness	Constriction (%)	Flatness (%)	Moisture content (%)	Moisture uptake (%)
**1**@AHSP	0.40	0	100	0.17	11.12
**2**@AHSP	0.42	0	100	0.47	7.49
AHSP	0.40	0	100	0.06	12.59

References

S1. Wang, W.; Pang, X.; Wang, H.; Wu, Q.; Zheng, L.; Xu, B.; Wang, J.; Shi, X.; Bai. F.; Liu, H. Bioactive Metal-Organic Frameworks with Specific Metal-Nitrogen (M-N) for Efficient Sondynamic Tumor Therapy. *ACS Nano*, **2021**, *15*, 20223-20012.

S2. Busato, M.; Melchior, A.; Migliorati, V.; Colella, A.; Persson, I.; Mancini,G.; Veclani, D.; D'Angelo, P. Elusive Coordination of the Ag^+ Ion in Aqueous Solution: Evidence for a Linear Structure, *Inorg. Chem*. **2020**, *59*, 17291−17302.

S3. Pluskal, T.; Castillo, S.; Villar-Briones, A.; Orešič, M. MZmine 2: Modular framework for processing, visualizing, and analyzing mass spectrometry-based molecular profile data, BMC Bioinformatics, **2010**, 11:395, PMID: 20650010.

S4. Jaros, S.W.; Krogul-Sobczak, A.; Bażanów, B.; Florek, M.; Poradowski, D.; Nesterov, D.S.; Śliwińska-Hill, U.; Kirillov, A.M.; Smoleński, P. Self-Assembly and Multifaceted Bioactivity of a Silver(I) Quinolinate Coordination Polymer. *Inorganic Chemistry*, **2021**, *60*, 15435–15444.

S5. Grove, D. C.; Randall, W. A. Assay Methods of Antibiotic. A Laboratory Manual; Medical

Encyclopedia: New York, **1955**.

S6. Li, X.; Yan, Y. *J. Mol. Struct.* **2015**, *1082*, 170-173.

S7. Lehrer, S. S. Solute perturbation of protein fluorescence. Quenching of the tryptophyl fluorescence of model compounds and of lysozyme by iodide ion, *Biochemistry,* **1971**, *10,* 3254-3263.

S8. Zhang, X. F.; Shu, C. Y.; Xie, L.; Wang, C. R.; Zhang, Y. Z.; Xiang, J. F.; Li, L.; Tang, Y. L. Protein conformation changes induced by a novel organophosphate-containing water-soluble derivative of a C60 fullerene nanoparticle, *J. Phys. Chem. C*, **2007**, *111*, 14327–14333.

S9. Lakowicz, J.R. Principles of Fluorescence Spectroscopy, 3rd ed., Springer, New York, **2006**.

S110. Neault, J.F.; Tajmir-Riahi, H.A. Interaction of cisplatin with human serum albumin. Drug binding mode and protein secondary structure. *Biochim. Biophys. Acta*. **1998**, *1384,* 153–159

S11. Domotor, O.; Hartinger, C. G.; Bytzek, A. K.; Kiss, T.; Keppler, B. K.; Enyedy, E. A.; Characterization of the binding sites of the anticancer ruthenium(III) complexes KP1019 and KP1339 on human serum albumin via competition studies, *J. Biol. Inorg. Chem.* **2013**, *18*, 9–17

S12. Kelly, S. M.; Jess, T. J.; Price, N. C. Haw to study proteins by circular dichroism, *Biochim. Biophys. Acta*, **2005**, *1751*, 119-139

S13. Berman, H. M.; Westbrook, J.; Feng, Z.; Gilliland, G.; Bhat, T. N.; Weissig, H.; Shindyalov, I. N.; Bourne, P. E. *Nucl. Acids Res.* **2000**, *28*, 235–242.

S14. Morris, G. M.; Huey, R.; Lindstrom, W.; Sanner, M. F.; Belew, R. K.; Goodsell, D. S.; Olson, A. J. *J. Comput. Chem.* **2009**, *30*, 2785–2791.

S15. Trott, O.; Olson, A. J. *J. Comput. Chem.* **2010**, *31*, 455–461.

S16. Haq. A.; Kumar, S.; Mao, Y.; Berthiaume, F.; Michniak-Kohn, B. Thymoquinone-Loaded Polymeric Films and Hydrogels for Bacterial Disinfection and Wound Healing. Biomedicine, **2022**, *8*, 386.
